# Deformable nanocarriers for enhanced drug delivery and cancer therapy

**DOI:** 10.1002/EXP.20230037

**Published:** 2024-03-15

**Authors:** Ziyang Cao, Jing Liu, Xianzhu Yang

**Affiliations:** ^1^ Department of General Surgery Guangzhou First People's Hospital the Second Affiliated Hospital South China University of Technology Guangzhou People's Republic of China; ^2^ Center for Medical Research on Innovation and Translation Institute of Clinical Medicine School of Medicine Guangzhou First People's Hospital South China University of Technology Guangzhou People's Republic of China; ^3^ School of Chemistry Chemical Engineering and Biotechnology Nanyang Technological University Singapore Singapore; ^4^ School of Biomedical Sciences and Engineering South China University of Technology Guangzhou International Campus Guangzhou Guangdong People's Republic of China

**Keywords:** cancer therapy, deformable nanocarriers, drug delivery, stimuli‐responsive

## Abstract

Recently, the field of nanomedicine has witnessed substantial advancements in the development of nanocarriers for targeted drug delivery, emerges as promising platforms to enhance therapeutic efficacy and minimize adverse effects associated with conventional chemotherapy. Notably, deformable nanocarriers have garnered considerable attention due to their unique capabilities of size changeable, tumor‐specific aggregation, stimuli‐triggered disintegration, and morphological transformations. These deformable nanocarriers present significant opportunities for revolutionizing drug delivery strategies, by responding to specific stimuli or environmental cues, enabling achieved various functions at the tumor site, including size‐shrinkage nanocarriers enhance drug penetration, aggregative nanocarriers enhance retention effect, disintegrating nanocarriers enable controlled drug release, and shape‐changing nanocarriers improve cellular uptake, allowing for personalized treatment approaches and combination therapies. This review provides an overview of recent developments and applications of deformable nanocarriers for enhancing tumor therapy, underscores the diverse design strategies employed to create deformable nanocarriers and elucidates their remarkable potential in targeted tumor therapy.

## Graphical abstract

Deformable nanocarriers with various morphological transformations for diferent potential applications.

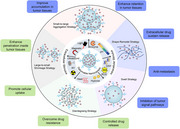

## 1 INTRODUCTION

In recent years, significant advancements have been made in the development of nanocarriers for drug delivery, enhancing the delivery efficiency and efficacy of therapeutic agents.^[^ ^1^, ^2^, ^3^, ^4^ ^]^ Nanoparticles, liposomes, micelles, and dendrimers have emerged as promising nanocarriers, providing opportunities for targeted drug delivery and controlled release.^[^ ^5^, ^6^, ^7^, ^8^ ^]^ However, despite these advancements, drug delivery faces several physiological barriers and challenges for in vivo delivery, including biological barriers, tumor accumulation and retention, stability and drug release, etc., substantially restricting the therapeutic efficacy of nanomedicines. Recently, Wilhelm et al. analyzed 117 published preclinical studies and reported that only 0.7% of the injected dose of NP‐based drugs was delivered to the solid tumor, which is somewhat unexpected and disappointing.^[^ ^9^ ^]^ In contrast, meta‐analyses performed by Lauren et al. demonstrated that the overall exposure of nano‐based drugs to a tumor was 76.12% that of the overall plasma exposure, which is a promising result.^[^ ^10^ ^]^ Therefore, while the majority of nanomedicines systemically administered can be exposed to tumor tissues, only a small fraction of nanomedicines can accumulate and exert therapeutic efficacy at the tumor site. Regarding this issue, scientists are attempting to design multifunctional stimuli‐responsive nanocarriers, aiming to overcome the physiological barriers and enhance the efficiency of one or multiple processes involved in the delivery of nanomedicines in vivo, including prolonged drug circulation, enhanced accumulation and retention in tumor site, improved tumor penetration capability, and even rapid drug release at tumor sites, in order to achieve effective tumor therapeutic outcomes.^[^ ^11^, ^12^ ^]^

Recently, the field of stimulus‐responsive nanocarriers has witnessed significant development. Stimuli such as changes in pH, temperature, enzymes, or redox conditions were engineered into nanocarriers to trigger drug release or modulate their properties.^[^ ^13^, ^14^ ^]^ This approach offers enhanced spatiotemporal control over drug delivery, allowing for site‐specific and on‐demand release of therapeutic agents, including pH‐responsive nanoparticles, thermo‐responsive polymers, enzyme‐responsive liposomes, and redox‐responsive nanocarriers. These nanocarriers are typically engineered using smart materials that undergo changes in their physicochemical properties in response to external or internal stimuli, for targeted drug delivery, improved therapeutic efficacy, and reduced off‐target effects,^[^ ^15^ ^]^ which have demonstrated promising applications in cancer therapy, infectious diseases, and regenerative medicine. Additionally, the integration of multiple stimulus‐responsive mechanisms has led to the development of multifunctional nanocarriers with enhanced capabilities, such as dual or triple stimuli responsiveness.

Among them, deformable nanocarriers have emerged as a promising class of drug delivery systems with significant advantages and versatile applications.^[^ ^16^, ^17^ ^]^ Deformable nanocarriers refer to nanoscale carriers capable of undergoing structural changes in response to ex/internal stimuli or physiological conditions. This deformability enables them to adapt their shape, size, and surface properties, thereby enhancing their interactions with biological barriers and improving drug delivery efficacy.^[^ ^18^, ^19^ ^]^ For example, deformable nanocarriers can reshape and navigate through constricted physiological barriers like the extracellular matrix or the blood‐brain barrier.^[^ ^20^ ^]^ This adaptability allows for superior tissue penetration and targeted accumulation.^[^ ^21^ ^]^ Additionally, they can react to specific environmental triggers, ensuring controlled drug release at the desired location and granting precise temporal control over drug delivery.^[^ ^22^ ^]^ These nanocarriers also could promote efficient cellular internalization upon interaction with cell membranes and facilitate endosomal escape,^[^ ^23^, ^24^ ^]^ and enabling targeted drug delivery and reduced off‐target effects.^[^ ^25^, ^26^ ^]^ Among all the strategies, designing nanocarriers with tunable sizes is the most intuitive and controllable approach, including enhanced tissue penetration, controlled drug release, improved cellular uptake, promote cellular uptake, anti‐metastasis, etc., making them versatile drug delivery platforms with broad applications in diverse therapeutic areas.

**FIGURE 1:**
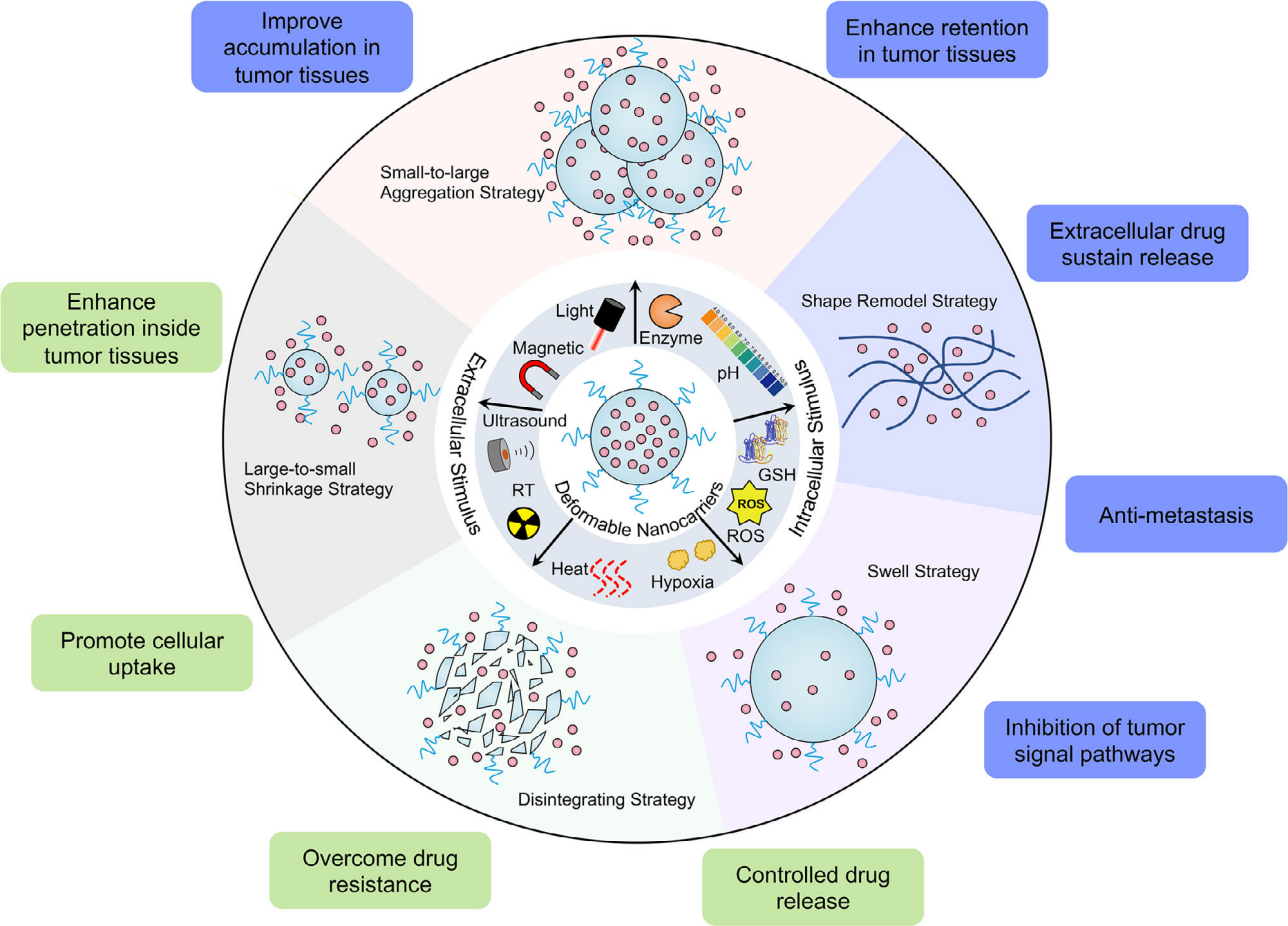
Brief illustration of stimuli‐triggered deformable nanocarriers with various morphological transformations for various potential applications.

In this review, we will summarize intelligent deformable nanocarriers‐designed strategies, including size‐aggregating nanocarriers,^[^ ^27^, ^28^ ^]^ size‐shrinkage nanocarriers,^[^ ^29^, ^30^ ^]^ shape‐changing nanocarriers ^[^ ^31^, ^32^ ^]^ and disintegrating nanocarriers ^[^ ^33^, ^34^ ^]^ based on ex/intracellular stimulus, as well as their potential applications (Figure 1). Each section involves nanocarriers deformation processes triggered by different stimuli such as pH, enzyme, redox, light, temperature, etc. In order to clarify the enhanced antitumor efficiency of deformable nanocarriers, we focus on the improving drug delivery efficiency for tumor therapy. Aggregation strategies and shape‐changing strategies can be used in enhanced drug accumulation and retention, shrinkage strategies can improve tumor tissue penetration, while disintegration exhibit advantages in triggering drug release. In the end, we have presented an outlook on the utilization of deformable nanocarriers for tumor therapy and deliberated on the forthcoming challenges in their future applications.

## 2 PHYSICOCHEMICAL PROPERIES OF NANOCARRIERS IMPACT ON DRUG DELIVERY EFFICACY

The size, morphology, and other physicochemical properties of nanocarriers significantly influence in vivo drug delivery efficiency.^[^ ^35^, ^36^, ^37^ ^]^ The rational design and engineering of nanocarriers with specific size and shape allow for enhanced interactions with biological barriers, targeted tissue accumulation, and controlled drug release at the desired site.^[^ ^38^, ^39^, ^40^ ^]^ The size of nanocarriers is a crucial determinant of their in vivo behavior. Nanocarriers with appropriate sizes (typically in the range of 10–200 nm) exhibit prolonged blood circulation and reduced reticuloendothelial system (RES) uptake, leading to enhanced passive targeting to tumor tissues through the enhanced permeability and retention (EPR) effect.^[^ ^41^ ^]^ Moreover, smaller nanocarriers can extravasate more easily and penetrate tumor tissues,^[^ ^42^ ^]^ whereas larger nanocarriers may have an extended circulation half‐life,^[^ ^43^ ^]^ allowing for increased accumulation at the target site. On the other hand, the morphology of nanocarriers, such as spherical, rod‐shaped, or discoidal, impacts their interactions with biological components.^[^ ^44^ ^]^ Non‐spherical nanocarriers may offer improved cellular internalization due to their higher aspect ratios and increased contact area with cell membranes.^[^ ^45^ ^]^ Additionally, certain shapes can exploit specific biological processes for targeted drug delivery, enhancing drug delivery efficiency.^[^ ^46^, ^47^ ^]^

Surface charge also plays a critical role in determining the interactions between nanocarriers and biological entities.^[^ ^48^ ^]^ Neutral or slightly negatively charged nanocarriers generally exhibit reduced opsonization and prolonged circulation time in vivo.^[^ ^49^ ^]^ Surface coating with biocompatible materials, such as polyethylene glycol (PEG), can further shield nanocarriers from recognition by the reticuloendothelial system (RES), thus promoting evasion of phagocytic clearance and increasing drug delivery efficiency.^[^ ^50^, ^51^ ^]^ Nanocarriers also could achieve active targeting after incorporating targeting ligands on the surface for the purpose of binding to specific cell receptors overexpressed in tumor tissues.^[^ ^52^, ^53^ ^]^ Nanocarriers with active targeting could enhance nanocarrier‐cell interactions, leading to improved cellular internalization and drug delivery efficiency, resulting in improved antitumor efficacy. Hence, the rational manipulation of nanocarrier size, morphology, surface charge, and surface functionalization are essential for optimizing drug delivery efficiency and achieving satisfying therapeutic outcomes. Understanding how these physicochemical properties impact the behavior of nanocarriers in vivo can guide the design of tailored drug delivery systems with enhanced therapeutic efficacy and reduced systemic toxicity.

Deformable nanocarriers offer distinct advantages of variable physicochemical properties upon stimulus‐responsive in vitro or in vivo for antitumor drug delivery, making them as promising candidates for enhancing therapeutic outcomes in cancer treatment.^[^ ^54^, ^55^, ^56^ ^]^ Deformable nanocarriers can undergo structural changes in response to the tumor microenvironment, such as aggregation, size‐shrinkage, disintegration or shape transformation. These properties could improve tumor penetration, enhance cellular uptake, achieve active targeting or sustained drug release, reduce systemic toxicity, and enable combination therapy and therapeutic application.^[^ ^57^, ^58^, ^59^, ^60^ ^]^ Overall, deformable nanocarriers offer a versatile platform for antitumor drug delivery, addressing challenges associated with conventional drug delivery approaches. Their ability to adapt to the dynamic tumor microenvironment and effectively deliver therapeutic agents holds great promise for the advancement of cancer treatment strategies with enhanced efficacy and reduced toxicity.

## 3 SMALL‐TO‐LARGE AGGREGATION STRATEGY

Statistical analysis from the past few decades in the realm of nanocarrier‐based drug delivery reveals that while about 70% of nanocarriers can reach the tumor site after systemic administration,^[^ ^10^ ^]^ only a small percentage (around 0.7%, median) actually settle and accumulate at there.^[^ ^9^ ^]^ The limited accumulation efficiency impedes the therapeutic effectiveness of nanomedicines administered systemically. Thus, boosting the intratumoral retention of nanomedicines that arrive at the tumor site is crucial for enhancing their overall therapeutic efficacy.^[^ ^61^ ^]^ Large‐sized nanocarriers typically exhibit prolonged retention in the intricate vascular microenvironment of tumor tissues. Consequently, researchers have proposed an aggregation strategy to craft tumor stimulus‐responsive nanocarriers for drug delivery. This approach allows the nanocarriers to transition of nanocarriers from a small size to a larger size at the tumor site, thereby enhancing retention. Given the heterogeneity of tumors, the stimuli prompting this change are multitudinous, encompassing factors like a mildly acidic microenvironment and highly expressed enzymes,^[^ ^13^ ^]^ etc. Moreover, intelligent size‐aggregating nanocarriers also could be designed based on external stimuli, such as light and temperature.^[^ ^62^ ^]^ In response to these external/internal stimuli, initial nanoparticles with relatively small sizes undergo interactions, including click reactions, self‐assembly, electrostatic interactions, or phase transitions, leading to the nanocarrier aggregations and enhanced tumor retention.

### 3.1 pH‐responsive crosslinking nanocarriers

The tumor microenvironment tends to be mildly acidic, with a pH of approximately 6.5, due to the increased glycolysis of tumor cells.^[^ ^63^, ^64^ ^]^ Once internalized into cells, nanocarriers will enter into endosomes or lysosomes, which display an even more acidic environment with pH levels ranging from 5.0 to 5.5.^[^ ^65^ ^]^ In contrast, blood and normal tissues consistently maintain a pH of 7.4.^[^ ^66^ ^]^ Leveraging the pH disparity between normal and tumor tissues, the design of pH‐responsive deformable nanocarriers for antitumor drug delivery shows potential in facilitating the aggregation of small‐sized nanoparticles and their transformation into larger aggregations within the extracellular tumor microenvironment.

**FIGURE 2:**
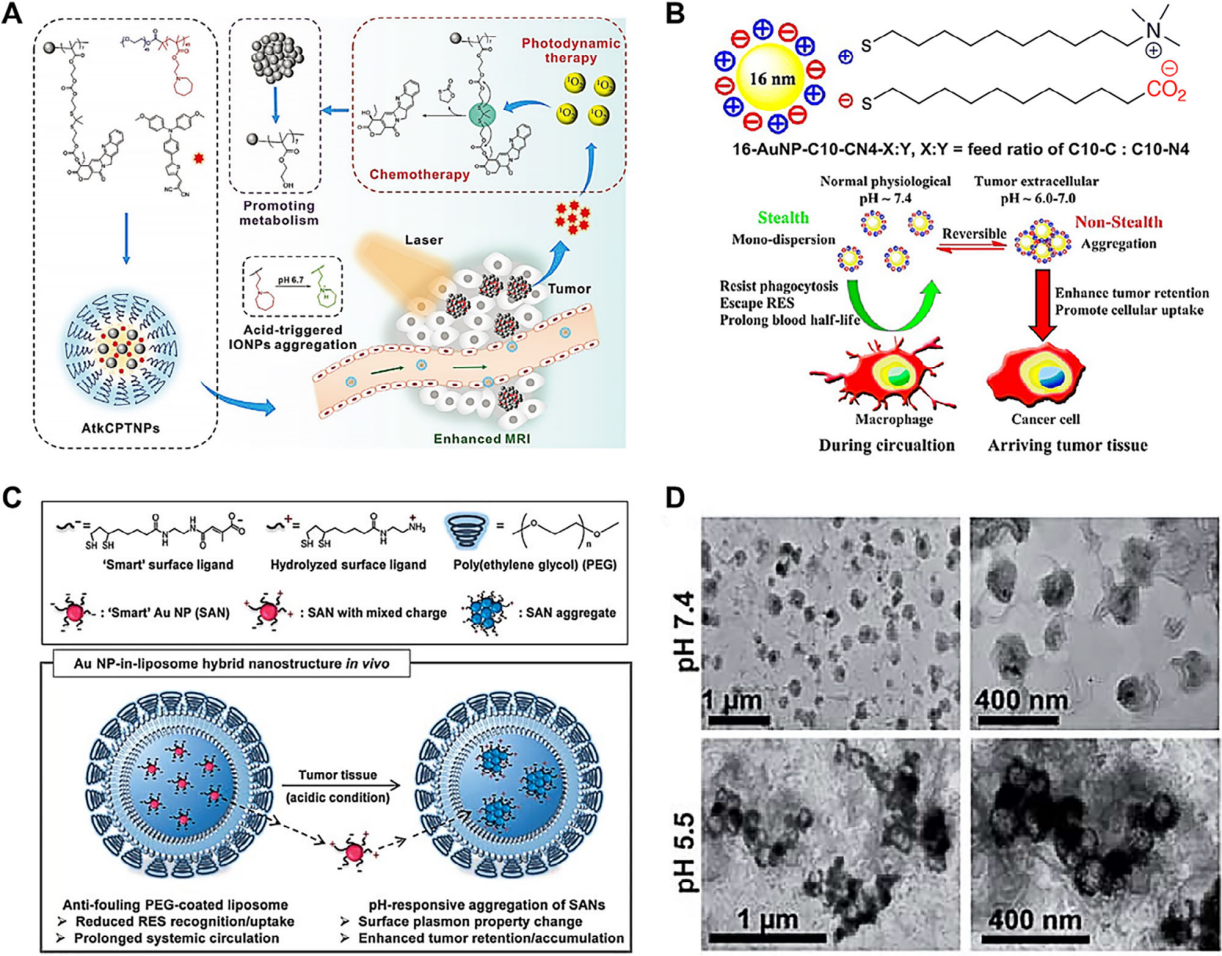
Acid‐triggered nanocarriers crosslinked strategies. (A) Schematic illustration of AtkCPTNPs for enhanced MRI‐guided chemo/photodynamic combination therapy. Reproduced with permission.^[^ ^67^ ^]^ Copyright 2021, John Wiley and Sons. (B) Schematic illustration of the hypothesis that retention and cellular uptake of small nanoparticles in tumors can be enhanced be inducing aggregation of nanoparticles in the tumor environment. Reproduced with permission.^[^ ^68^ ^]^ Copyright 2013, American Chemical Society. (C) Schematic illustration of a Au NPs‐in‐liposome hybrid nanostructure for systemic tumor delivery. pH‐responsive ‘smart’ SANs are encapsulated in a PEG‐grafted liposome which greatly reduces RES uptake and improves blood circulation/tumor permeation of SANs, and could occur pH‐responsive aggregation upon exposure to the acidic conditions in tumors with largely increased tumor accumulation levels. (D) Transmission electron microscope (TEM) images of SANLIPO measured after 2% uranyl acetate staining; low (left) and high (right) magnification images after 180 min in pH 7.4 (top) or in pH 5.5 (bottom). Reproduced with permission.^[^ ^69^ ^]^ Copyright 2013, the Royal Society of Chemistry.

Based on the weakly acidic tumor environment, Wang et al. introduced a nanotheranostic agent (AtkCPTNPs), which could engineer to undergo a two‐stage programmed size alteration, optimizing magnetic resonance imaging (MRI)‐guided chemo/photodynamic combination therapy.^[^ ^67^ ^]^ The AtkCPTNPs were synthesized by assembling pH‐responsive amphiphilic poly(ethylene glycol)‐poly(2‐(hexamethyleneimino) ethyl methacrylate) (PEG‐PC7A), reactive oxygen species (ROS)‐sensitive poly camptothecin (CPT) prodrug‐modified iron oxide nanoparticles (IONPs), and an aggregation‐induced emission (AIE) photosensitizer, MeOTTMN. Owing to their nanoscale dimensions, AtkCPTNPs could efficiently target tumor sites via passive targeting mechanisms. Within the acidic milieu of the tumor microenvironment, the hydrophobic‐to‐hydrophilic transition of PC7A triggered by pH, coupled with the release of hydrophobic polyprodrug‐modified IONPs, led to the formation of substantial IONP aggregates (Figure 2A). This aggregation facilitated enhanced tumor retention and augments MR signal intensity. Subsequently, under MRI guidance, the tumor region is exposed to white light, prompting MeOTTMN to generate a significant quantity of ROS. This served for two purposes: facilitating photodynamic therapy (PDT) and instigating controlled CPT release, achieving a synergistic chemo/photodynamic therapeutic effect. Notably, post the exhaustion of diagnostic and drug delivery functionalities, the large aggregates reverted to monodispersed IONPs, and promoted a faster metabolic clearance rate for IONPs, mitigating potential toxicity risks associated with prolonged retention. These findings presented a novel strategy of programmed size modulation in nanocarriers, striking a balance between targeted therapeutic efficacy and biosafety considerations.

Liu et al. focus on synthesis a pH‐responsive zwitterionic gold nanoparticles (AuNPs), which exhibited a pronounced resistance to phagocytosis by macrophages under normal pH conditions and aggregated at tumor acidic environment for enhanced retention (Figure 2B).^[^ ^68^ ^]^ These AuNPs were functionalized using a combination of self‐assembled monolayers (SAMs) comprising weak electrolytic 11‐mercaptoundecanoic acid and the strong electrolytic (10‐mercaptodecyl)trimethylammonium bromide. This modification strategy facilitated the generation of mixed‐charge zwitterionic AuNPs that exhibited desired pH sensitivity, making them suitable for targeting acidic tumor microenvironments and occurring aggregation induced deformation. These AuNPs nanoparticles (≈20 nm) exhibited excellent dispersion within the pH range typical of blood and normal tissues, being attributed to their non‐adhesive zwitterionic nature. However, these AuNPs could form aggregation (≈300 nm) under acidic tumor environment according to van der Waals attraction, hydrogen bonding, electrostatic repulsion, hydration repulsion, and other forces. The formation of larger aggregates by the nanoparticles within tumor tissues is anticipated to enhance their retention within the tumor matrix and improve tumor internalization. Sungjee Kim et al. designed a pH‐responsive hybrid nanostructure composed of AuNPs and liposomes (SANLIPO), wherein pH‐sensitive “smart” gold nanoparticles (SANs) are encapsulated within PEGylated liposomes.^[^ ^69^ ^]^ This hybrid configuration synergistically integrates the pH‐responsive assembly and plasmonic property alterations of SANs with the enhanced systemic circulation and tumor targeting capabilities of PEGylated liposomes (Figure 2C). The anti‐fouling PEG‐coated liposome could reduce RES recognition/uptake and prolong systemic circulation. While the liposome reached tumor tissue with acidic condition, the inner “Smart” SANs occurred charge and surface plasmon property changes, and then aggregated to form larger SANs particles in meantime for enhanced tumor accumulation and retention (Figure 2D). This AuNPs‐liposome hybrid nanoparticles offers a novel platform for augmenting imaging and therapeutic efficacies by tailoring the pharmacological attributes of functional nanoparticles.

### 3.2 Enzyme‐induced aggregating nanocarriers

Within the tumor microenvironment, specific enzymes are overexpressed due to tumor heterogeneity, genetic mutations, and cell proliferation.^[^ ^70^, ^71^ ^]^ Tumor cells interact with surrounding cells via cytokines and growth factors, leading to the upregulation of enzymes such as matrix metalloproteinases, tyrosine kinases, cyclooxygenases, acid hydrolases, telomerase, and thymidylate synthase, etc. These enzymes critically influence tumor growth, invasion, metastasis, and treatment outcomes.^[^ ^72^ ^]^ Moreover, tumor hypoxia and inflammatory conditions further induce enzyme expression. Utilizing these overexpressed enzymes to design enzyme‐induced deformable nanocarriers can optimize drug delivery and enhance antitumor effects.

**FIGURE 3:**
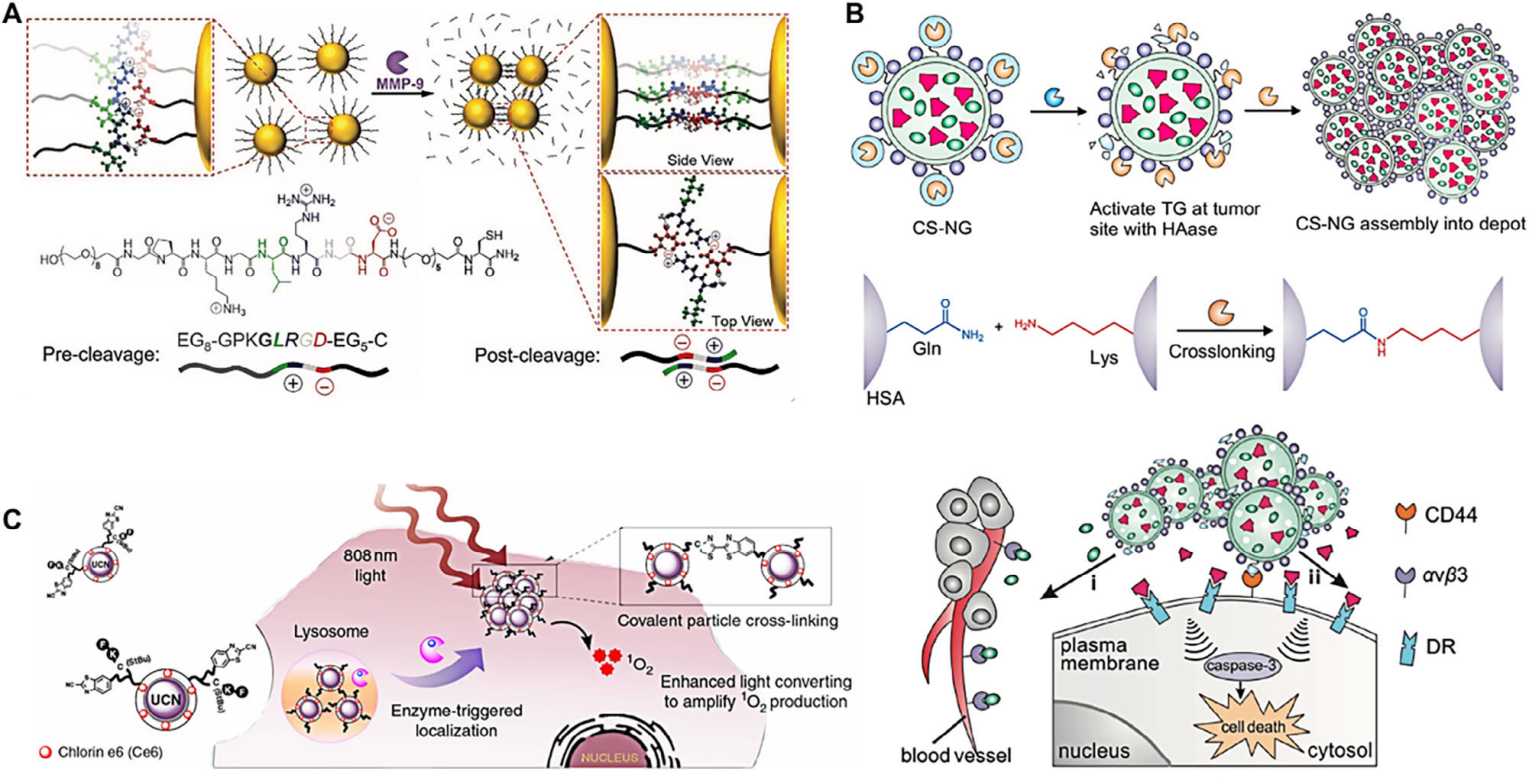
Enzyme‐triggered nanocarriers crosslinked strategies. (A) Schematic illustration of MMP‐9‐triggered assembly crosslinking of AuNPs using charge‐complementary peptides. Reproduced with permission.^[^ ^73^ ^]^ Copyright 2022, John Wiley and Sons. (B) Schematic of tumor microenvironment‐mediated construction of combination drug‐delivery depots for sustained drug release using CS‐NG. Reproduced with permission.^[^ ^74^ ^]^ Copyright 2016, American Chemical Society. (C) Illustration of the microenvironment‐sensitive strategy for covalent cross‐linking of peptide‐premodified UCNs in tumor areas. Reproduced with permission.^[^ ^75^ ^]^ Copyright 2016, Springer Nature.

Ulijn et al. designed a gold nanoparticle carrier based on zwitterionic peptides that were responsive to matrix metalloproteinase‐9 (MMP‐9), enabling enzyme‐triggered assembly and aggregation at tumor sites (Figure 3A).^[^ ^73^ ^]^ Zwitterions is one molecule possessing both positive and negative charges, offering unique interactions and self‐assembly behaviors in nanoparticle design. This self‐assembly is activated by the enzymatic release of surface‐bound zwitterionic tetrapeptides in the presence of MMP‐9, which is overexpressed in cancer cells. Through multivalent and self‐complementary interactions of the zwitterionic tetrapeptides, a robust nanoparticle assembly is achieved, directing the self‐assembly of gold nanoparticles. Based on the overexpressed MMP‐9 of cancer cells, the process of nanoparticle assembly process occurs near the cell membrane, leading to a size‐induced selection of cellular uptake mechanisms, resulting in diminished cell growth. The authors provided confocal images of cancer cells treated with different nanoparticles, highlighting the distinct cellular uptake mechanism. Additionally, TEM images showed the distinct features of macropinocytosis versus receptor‐mediated endocytosis in treated cancer cells. This assembly process directly influenced the cellular uptake mechanism of nanoparticles, and altered the uptake in tumor cells, thus influencing cell growth. Furthermore, the enzyme responsiveness and uptake route of the system can be programmed by customizing the peptide sequence. This design permits enzyme‐triggered assembly and offers a customizable approach for programming nanoparticle behavior in a biological context. It presents novel avenues and potential for further applications of nanoparticle carriers, especially in targeted drug delivery for specific cells or disease states.

In a pioneering study, Gu et al. engineered a tumor microenvironment‐responsive CS‐NG nanocarrier system, which is sensitive to hyaluronidase (HAase, an overexpressed enzyme at the tumor microenvironment) and can enzymatically assemble into microsized extracellular depots for extracellular drug delivery and sustain release (Figure 3B).^[^ ^74^ ^]^ This innovative system exhibits a unique “morphing aggregation” behavior specifically at tumor sites by transglutaminase (TG) catalyzes the crosslinking of glutamine (Gln) and lysine (Lys) on human serum albumin (HAS) surface of CS‐NG nanocarrier to form depots. Such “deformable” nanocarriers demonstrate a heightened selective responsiveness within the tumor milieu, facilitating the formation of extracellular drug depots for the sustained release of therapeutics targeting membrane‐associated sites. In the study, TRAIL (apoptosis inducing ligand, a paramount extracellular activator for inducing apoptosis) and cilengitide (a cyclic RGD pentapeptide exhibiting inhibitory effects on various tumors) were employed as model combination drugs. Cellular assays revealed that CS‐NG treated with HAase displayed a markedly enhanced cytotoxicity, with its IC_50_ value reduced by 3.2‐fold compared to the untreated CS‐NG. In cell viability assays involving human umbilical vein endothelial cells (HUVEC), both the native cilengitide and the cilengitide released from CS‐NG showed no significant difference in their effects on HUVEC cell viability. Moreover, cilengitide demonstrated cytotoxicity against MDA‐MB‐231 cells, and when combined with TRAIL, a synergistic anti‐cancer effect was observed. In tube formation assays, the released cilengitide exhibited pronounced inhibition of angiogenesis, an effect that was amplified with increasing concentrations of the released cilengitide. This research introduces a novel tumor microenvironment‐responsive nanocarrier system capable of specific “morphing” behavior at tumor sites, facilitating the sustained release of therapeutics targeting membrane‐associated sites. Overall, this work presents a novel approach that nanocarriers are designed to aggregate into larger structures in the presence of specific enzymes in the tumor microenvironment. This aggregation forms drug‐delivery depots, being able to enhance the delivery and efficacy of anticancer drugs.

Xing and his colleagues have designed a nanocarrier predicated on the efficient click reaction between cysteine (Cys) and 2‐cyanobenzothiazole (CBT). This enzyme‐responsive nanocarrier is tailored for the covalent cross‐linking aggregation of peptide‐modified upconversion nanocrystals (UCNs) at tumor sites.^[^ ^75^ ^]^ Upon arrival at the tumor location after intravenous (i.v.) injection, the surface peptide chains of UCNs were cleaved by the tumor‐specifically overexpressed enzyme, cathepsin B (CtsB). This cleavage process could re‐expose the free cysteine residues, which then engage in a click reaction with the CBT moieties on adjacent particles, culminating in inter‐nanoparticle covalent crosslinking (Figure 3C). This mechanism enhanced the accumulation and retention of UCNs within the tumor. Moreover, this enzyme‐induced covalent crosslinking augmented the upconversion emission of UCNs upon 808 nm laser irradiation, which amplified the generation of singlet oxygen from photosensitizers attached to the UCNs, further enhancing the anti‐tumor efficacy. This innovative approach offers promising prospects for enhanced photodynamic therapy both in vitro and in vivo.

### 3.3 Light‐triggered aggregation

Compared to endogenous stimuli, designing nanocarriers using external light as a triggering factor offers numerous advantages.^[^ ^76^ ^]^ These include precise spatiotemporal control over therapy, non‐invasiveness, and tunability, which collectively reduce systemic toxicity and enhance therapeutic efficiency. Utilizing external light to design smart nanocarriers presents a powerful tool for nanomedicine and drug delivery, boasting a solid research foundation and vast potential for application.

**FIGURE 4:**
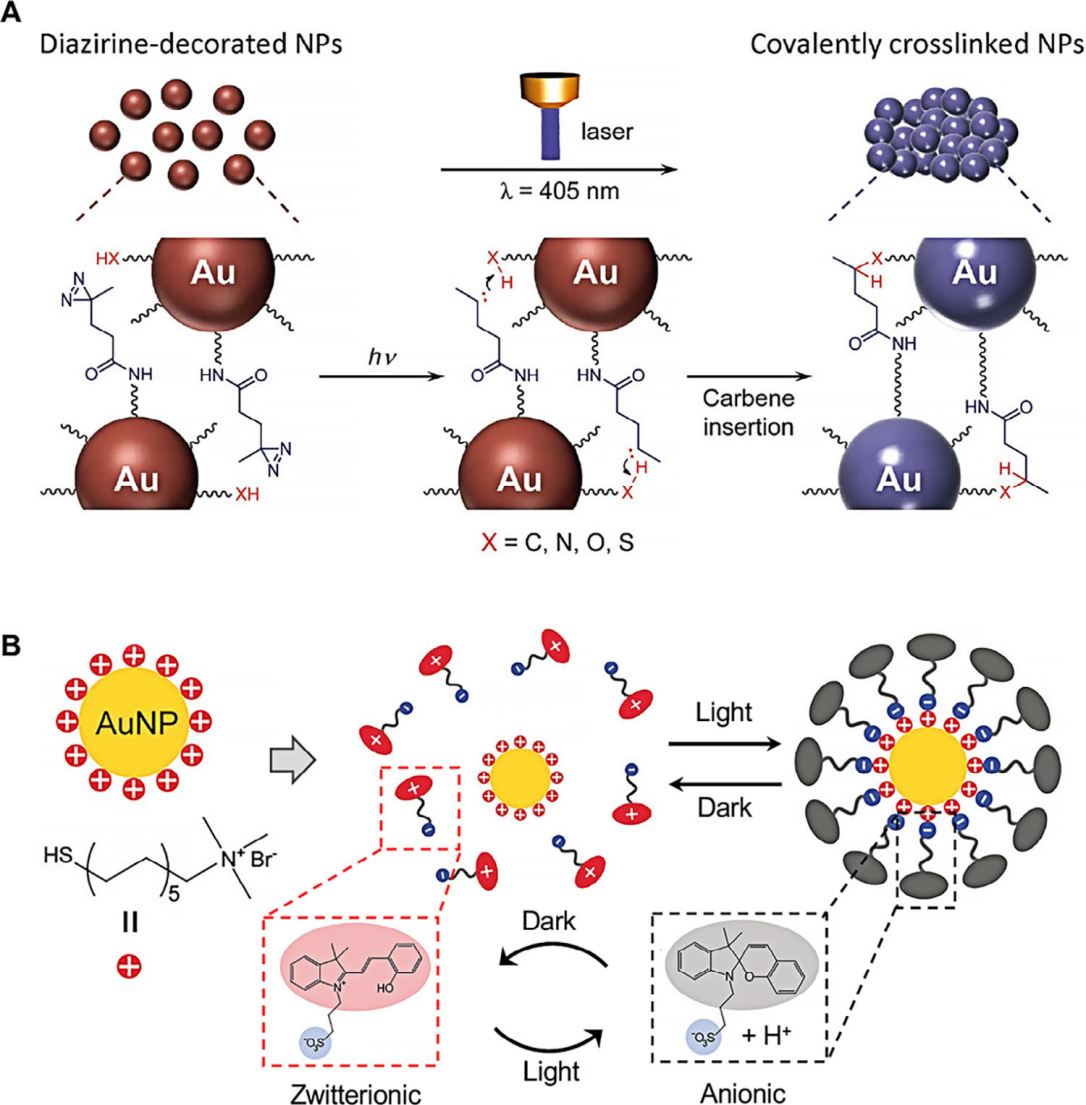
Light‐triggered nanocarriers crosslinked strategies. (A) Schematic illustration of light‐triggered assembly of dAuNPs. Reproduced with permission.^[^ ^77^ ^]^ Copyright 2017, John Wiley and Sons. (B) Light‐induced hydrophobization of cationic gold nanoparticles. Reversible switching could occur between the zwitterionic form and anionic form of the photoacid under irradiation. Then, the cationic AuNPs could adsorb the anionic photoacid on the cationic AuNPs. Reproduced under the terms of the CC‐BY‐NC‐ND.^[^ ^78^ ^]^ Copyright 2019, The Authors, published by the Royal Society of Chemistry.

Recently, many research groups have dedicated their efforts to developing nanocarrier system that undergo aggregation and morphological changes in response to light modulation. Gao and colleagues developed a photosensitive gold nanoparticle capable of covalent cross‐linking upon exposure to 405 nm laser irradiation.^[^ ^77^ ^]^ Upon this laser stimulation, the diazirine (DA) moieties on the nanoparticle surface undergo a transformation to carbene, facilitating the formation of covalent bonds with adjacent gold nanoparticles, leading to aggregation (Figure 4A). This in situ covalent aggregation of the gold nanoparticles shifted their surface plasmon resonance peak to the near‐infrared region, enhancing both photoacoustic imaging efficiency and photothermal therapeutic efficacy against tumors. The aggregation behavior of these nanoparticles under laser irradiation was demonstrated through transmission electron microscopy, dynamic light scattering (DLS), and absorption spectroscopy, revealing an increase in size from an initial 20.5–346 nm after 25 min irradiation. This strategy introduced an innovative strategy that employs light‐triggered manipulation of gold nanoparticle aggregation, offering a potent platform for photothermal therapy and photoacoustic imaging of tumors.

Moreover, light‐induced reversible aggregation has also been investigated in other groups. Zhang et al. introduced a method for the light‐induced reversible hydrophobization of cationic AuNPs,^[^ ^78^ ^]^ led to a transient assembly of AuNPs in an aqueous phase, achieved through the electrostatic adsorption of an anionic photoacid (Figure 4B). This approach is based on a photoacid that can toggle between two states: the zwitterionic form and the anionic form. In the absence of light exposure, the photoacid predominantly exists in its zwitterionic state, not specifically interacting with the cationic AuNPs. However, upon illumination, the photoacid translated from the zwitterionic state to the anionic state, leading to the adsorption of anionic spiropyran onto the nanoparticle surface, thereby forming a hydrophobic shell. This strategy presents a novel approach to control the surface properties and self‐assembly of nanoparticles based on light control. The findings provided a new avenue for manipulating nanoparticles for various applications, especially in the realm of nanotechnology and materials science.

### 3.4 Redox‐responsive aggregation

The rapid proliferation and robust metabolism of tumor cells lead to the significant production of reactive oxygen species (ROS).^[^ ^79^ ^]^ To neutralize the elevated ROS, cells often overexpress a substantial amount of glutathione (GSH) to counteract the increased ROS, thereby maintaining a redox balance that regulates tumor growth, invasion, metastasis, and therapeutic response.^[^ ^80^ ^]^ Research data indicate that within tumor regions, especially intracellularly, GSH concentrations can reach 2–10 mm, whereas ranging from 2 to 20 µm in normal tissues.^[^ ^81^ ^]^ Notably, disulfide bonds, as a classic chemical linkage that responds breaking to GSH, are widely employed in designing redox‐responsive nanocarriers for the delivery of anti‐cancer drugs.

**FIGURE 5:**
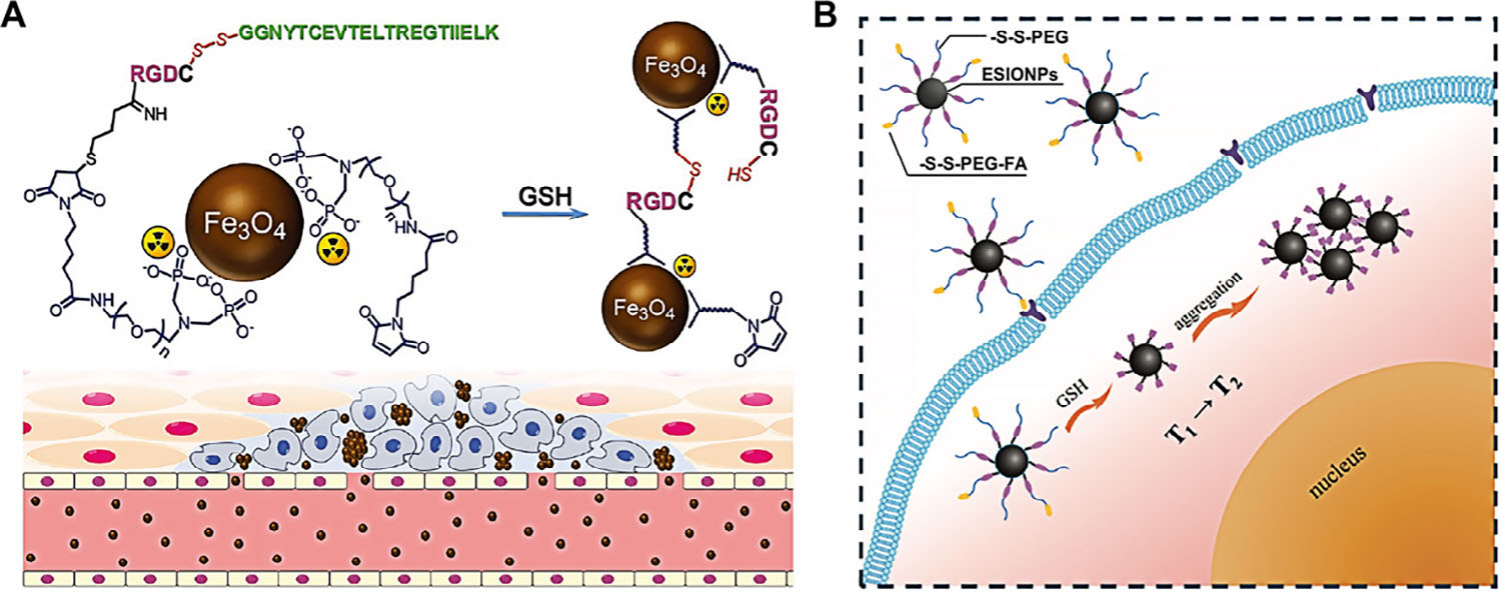
Redox‐triggered nanocarriers crosslinked strategies. (A) Schematic representation of ^99m^Tc‐labeled Fe_3_O_4_ nanoparticles designed for anti‐phagocytosis. These nanoparticles respond to GSH within the tumor microenvironment, leading to the formation of particle aggregates via interparticle crosslinking reactions. Reproduced with permission.^[^ ^82^ ^]^ Copyright 2017, John Wiley and Sons. (B) Schematic illustration of redox‐triggered aggregation of ESIONPs as T1/T2 switchable MRI agents for activating the T2 contrast effect. Reproduced with permission.^[^ ^83^ ^]^ Copyright 2021, the Royal Society of Chemistry.

Gao and colleagues developed a tumor microenvironment‐responsive nanoprobe for enhanced tumor imaging.^[^ ^82^ ^]^ This nanoprobe could effectively aggregate triggered by intracellular GSH in the tumor microenvironment, thereby improving the imaging sensitivity (Figure 5A). This nanoprobe used an asymmetric poly(ethylene glycol) (PEG) ligand and a tumor‐specific Arg‐Gly‐Asp peptide (RGD) for tumor targeting and a self‐peptide as a “mark of self ” linked through a disulfide bond. The self‐peptide acts as a stealth coating, ensuring efficient delivery of the nanoparticles to the tumor site. Once arrived at the tumor microenvironment, the disulfide bond was cleaved by GSH, achieving the nanoprobe anchor onto the surface of cancer cells upon the RGD. In meanwhile, the thiol group remaining on the RGD moiety then reacted with the maleimide group on the adjacent nanoparticle surface, resulting in nanoprobes effectively cross‐linking for enhanced tumor imaging. Based on the extremely rapidly reaction rate between sulfhydryl and maleimide, with the nanoparticles aggregated from 7.5–295 nm in 5 h. The research successfully demonstrated the potential of the GSH‐responsive anti‐phagocytosis ^99m^Tc‐labeled Fe_3_O_4_ nanoprobe in enhancing tumor imaging. By leveraging the unique properties of iron oxide nanoparticles and the characteristics of tumor microenvironment, the study provided a promising approach for precision tumor diagnosis.

Pei et al. presented a new redox‐triggered aggregation variant of ESIONP (enhanced superparamagnetic iron oxide nanoparticle) linked via a disulfide bond to PEG and end‐modified with folic acid (FA), termed ESIONPs‐s‐s‐PEG‐FA (Figure 5B).^[^ ^83^ ^]^ This design allowed for a tumor‐activatable T2 MRI contrast agent upon GSH‐induced aggregation of ESIONP. The ESIONPs‐s‐s‐PEG‐FA, when exposed to a reducing environment (like dithiothreitol, DTT), can effectively aggregate into clusters. This behavior was confirmed through TEM images and DLS measurements. The average hydrodynamic diameter of the non‐treated sample was approximately 12 nm, while the DTT‐treated sample showed a significantly larger size of about 342 nm. This redox‐responsive MRI contrast agent showed high specificity and selectivity in tumor locations. It also demonstrates negligible toxicity and favorable biocompatibility. The unique property of this agent to switch from T1 to T2 contrast in response to the tumor's redox environment makes it a promising candidate for designing specific T1/T2 switchable nanoplatforms for MRI applications.

### 3.5 Salt‐induced aggregation

As widely recognized, the in vivo fluid environment can be considered as a buffer salt system. However, a significant proportion of nanoparticles are inherently unstable and prone to undergo irreversible aggregation in high concentrations of aqueous salts, primarily due to the disruption of the shielding layer on their surface.^[^ ^84^ ^]^ To address this issue and extend the circulation time of nanocarriers in vivo, researchers have explored various strategies such as PEGylation and biomimetic cell membrane coating.^[^ ^85^, ^86^ ^]^

Sun et al. proposed a novel approach by leveraging the inherent instability of nanoparticles induced by salt.^[^ ^87^ ^]^ They designed salt‐induced aggregation of gold nanoparticles (GNPs) as a strategy for intratumoral injection. Upon intratumoral injection, GNPs rapidly formed irregular aggregations, while PEGylated GNPs remained unaggregated. The salt‐induced aggregation simplified surface modifications and occurred instantaneously. However, it should be noted that this strategy was only applied to superficial solid tumors and required precise injection due to the specific requirement of intratumoral delivery. This innovative approach represents a departure from conventional attempts to prolong nanocarrier circulation in vivo and offers an intriguing alternative for targeted drug delivery in specific tumor types. Nonetheless, its application is currently limited to superficial solid tumors and necessitates accurate delivery procedures.

## 4 LARGE‐TO‐SMALL SIZE CHANGE STRATEGY

The size of nanocarriers plays a significant role in their retention and penetration within tumor tissues. Nanocarriers with smaller size (e.g., less than 50 nm) tend to more readily penetrate deeper into tumor tissues, yet they can be easily pumped back into the bloodstream by the high interstitial fluid pressure of the tumor.^[^ ^88^ ^]^ In contrast, larger nanocarriers face challenges in penetration but are advantageous for retention at tumor sites.^[^ ^89^ ^]^ Furthermore, the tumor extracellular matrix presents a complex network where larger nanocarriers are likely to be hindered, while smaller particles can more easily traverse this extracellular matrix barrier.^[^ ^90^ ^]^ This suggests an opposite demand between the retention and penetration of nanocarriers, dependent on optimizing the nanocarriers size enhanced distribution in tumor tissues. Consequently, to strike a balance between the retention and penetration efficiency of nanocarriers in tumor sites, researchers have developed a series of size‐deformable nanocarriers to enhance both retention and penetration, resulting in improved anti‐tumor efficacy.^[^ ^91^ ^]^

### 4.1 Acidic pH‐triggered size shrinkage

**FIGURE 6:**
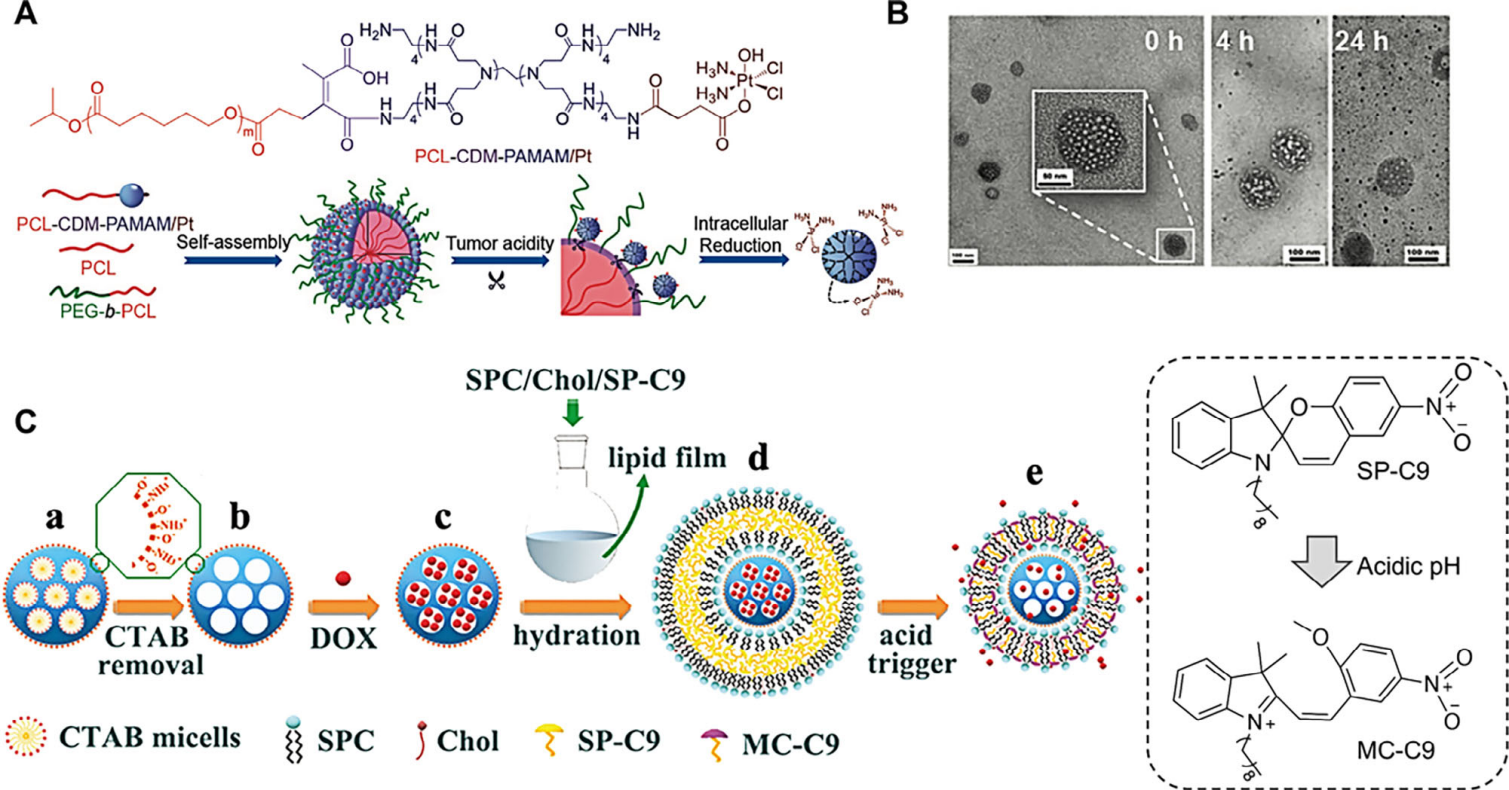
Acid‐triggered nanocarriers shrinkage strategies. (A) Preparation and physicochemical properties of the clustered nanoparticles based on the pH‐responsive PCL‐CDM‐PAMAM/Pt polymer and structural change of iCluster/Pt in response to tumor acidity and intracellular reductive environment. (B) TEM images of iCluster/Pt treated in PB at pH 6.8 for 0, 4, and 24 h, respectively (scale bar, 100 nm and for the inset images, 50 nm). Reproduced under the terms of the CC‐BY‐NC‐ND.^[^ ^95^ ^]^ Copyright 2016, The Authors, published by NAS. (C) Schematic preparation of USMSN‐NH_2_@LL‐SP‐C9 as a superior drug carrier with capacities of effective tumor accumulation and deep tumor penetration for high‐efficient cancer therapy. Upon acid triggering, hydrophobic SP‐C9 switched to amphiphilic MC‐C9, which induced tight re‐assembly of the alkyl chains of SPC and SP‐C9 with an accompanying decrease in the particle volume and drug release. Reproduced with permission.^[^ ^96^ ^]^ Copyright 2022, The Royal Society of Chemistry.

In the field of nanocarrier research, acid‐sensitive chemical bonds are frequently employed in the design of drug delivery systems, enabling the release of drugs in specific acidic microenvironments, thereby achieving promising antitumor efficacy.^[^ ^92^ ^]^ Common acid‐sensitive chemical bonds include ester bonds, acylhydrazone bonds, ketal and acetal bonds, ortho ester bonds, amide bonds, and lactone bonds.^[^ ^93^, ^94^ ^]^ These chemical bonds can be utilized in the design of acidic pH‐triggered size shrinkage nanocarriers. Li and colleagues engineered a tumor acidity‐responsive smart polymeric clustered nanoparticle, has an initial size of ≈100 nm termed iCluster, which was demonstrated to provide prolonged circulation in vivo.^[^ ^95^ ^]^ Upon the arrival at the acidic tumor microenvironment, the iCluster underwent an acid‐triggered release of smaller poly(amidoamine) (PAMAM) particles (≈5 nm), enhancing tumor penetration (Figure 6A). This transition of larger‐to‐smaller structural configurations significantly facilitated the permeation and internalization of therapeutic agents into tumors. In the simulated acidic environment in vitro, TEM observations revealed that iCluster could continuously release small‐sized particles (Figure 6B). The intrinsic design of iCluster allowed its physicochemical properties to adapt responsively to endogenous stimuli present within the tumor milieu and optimize therapeutic outcomes. In various challenging tumor models, iCluster had exhibited superior antitumor efficacy compared to other therapeutic modalities. The strategic design of iCluster paves the way for innovative advancements in next‐generation nanotherapeutics, representing a paradigm shift in nanoparticle‐based drug delivery.

Xu et al. developed a mesoporous silica‐based nanocarrier with size‐shrinking capabilities, enabling acid‐responsive contraction into smaller particles in the mildly acidic tumor microenvironment, thereby enhancing tumor penetration and therapeutic efficacy.^[^ ^96^ ^]^ This size‐reduction mechanism hinges on the acid‐sensitivity of 9‐alkyl‐spiropyran (SP‐C9). Under acidic conditions, SP‐C9 undergoes a transition from hydrophobic state to a hydrophilic form, 9 alkyl‐merocyanine (MC‐C9). This transformation prompts a rearrangement of the long alkyl chains within the lipid layer of the carrier, leading to a volume shrinkage of the lipid layer and a subsequent decrease in nanoparticle size, facilitating enhanced penetration (Figure 6C). Contact angle measurements revealed that SP‐C9 exhibited a contact angle of 143° in pH 7.4, which indicated its hydrophobic property. However, the contact angle reduced to 42° at pH 4.5, signifying the hydrophilic transformation of SP‐C9. This pH‐responsive triggered size‐shrinking nanocarrier presents a promising strategy for improved tumor penetration and therapeutic outcomes.

### 4.2 Enzyme‐triggered size shrinkage

**FIGURE 7:**
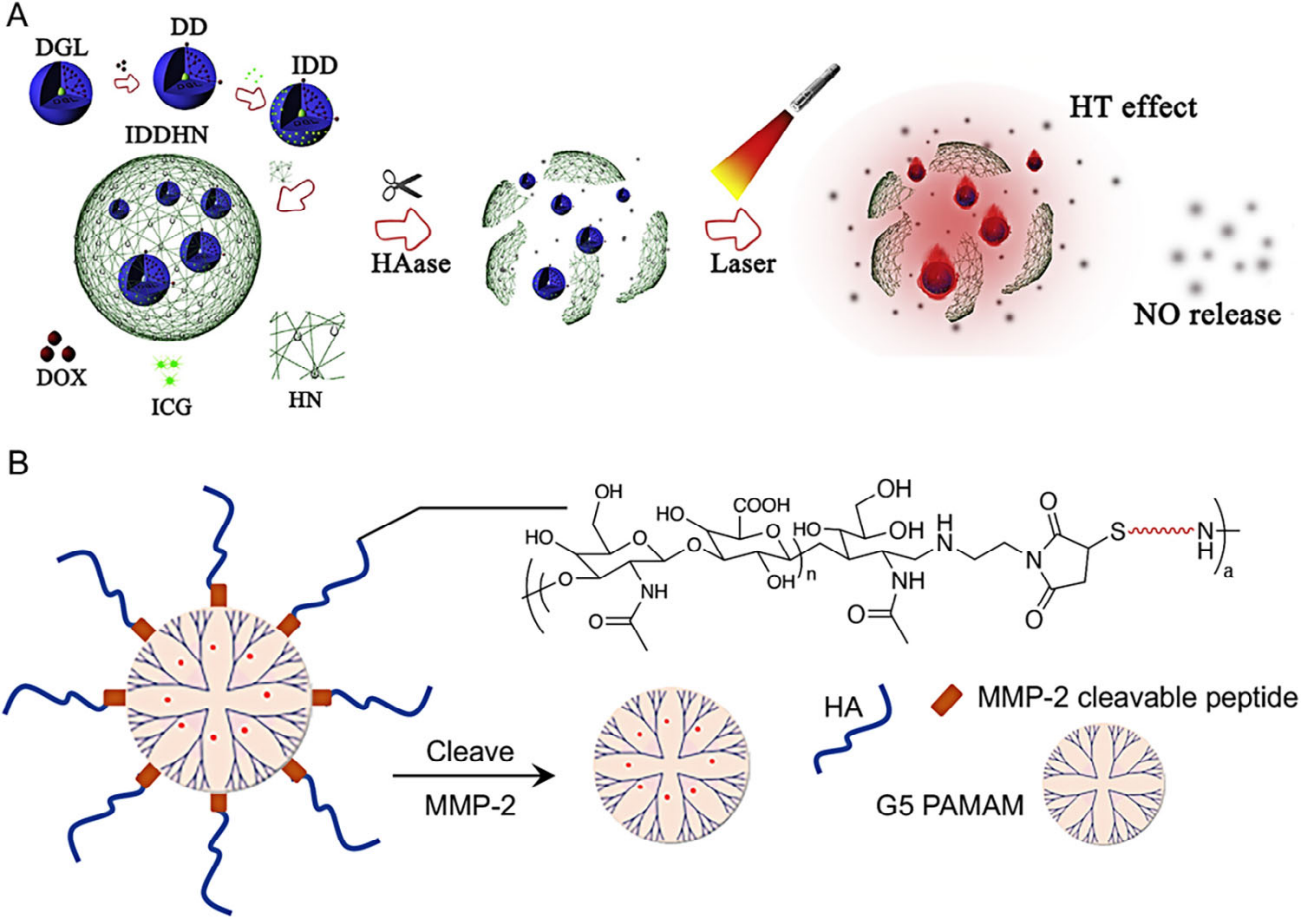
Enzyme‐triggered nanocarriers shrinkage strategies. (A) Schematic design of the IDDHN and release small particles under HAase degradation, and the synergistic effects of HT and NO could achieve under irradiation for deep tumor penetration and therapy effects. Reproduced with permission.^[^ ^97^ ^]^ Copyright 2018, Elsevier. (B) Schematic illustration to show the shrinkage of the HA‐pep‐PAMAM from 200 to 10 nm triggered by MMP‐2, a protease highly expressed in the tumor extracellular matrix. Reproduced with permission.^[^ ^98^ ^]^ Copyright 2017, American Chemical Society.

Utilizing enzymes that are specifically overexpressed in tumor tissues can also designed size‐shrinkable nanocarriers for enhanced tumor penetration and therapeutic efficacy. Gao et al. designed an enzyme‐responsive intelligent nanocarrier system, denoted as IDDHN, for synergistically enhancing tumor penetration.^[^ ^97^ ^]^ The IDDHN system is constructed by encapsulating small‐sized dendritic prodrug (IDD) and the photosensitizer indocyanine green (ICG) within a shell sensitive to near‐infrared (NIR) light, composed of a nitric oxide (NO) donor (HN) (Figure 7A). Upon reaching the tumor site, the HN shell of the IDDHN particles degraded by the abundant hyaluronidase (HAase) distributed in the tumor environment, leading to the release of the small‐sized IDD particles that could deeply penetrate the tumor tissue. Additionally, under external NIR irradiation, the encapsulated ICG generated heat, triggering the release of nitric oxide (NO), thereby synergistically enhancing both tumor penetration and therapeutic efficacy against the tumor.

Han and his colleagues proposed a size‐shrinkage drug delivery system based on a polysaccharide‐modified dendrimer with tumor microenvironment responsiveness, formed by conjugating the terminal glucose of hyaluronic acid (HA) to the superficial amidogen of poly(amidoamine) (PAMAM), using a matrix metalloproteinase‐2 (MMP‐2)‐cleavable peptide (PLGLAG) via click reaction^[^ ^98^ ^]^ (Figure 7B). The design strategy of this nanocarrier is harnessed the proteinase MMP‐2, which is highly expressed in the tumor extracellular matrix, to trigger the size‐shrinkage of HA‐pep‐PAMAM from 200 to 10 nm because of cleavage of peptide linker (PLGLAG), thereby facilitating penetration into deep tumor tissues. DLS tests indicate that co‐incubated HA‐pep‐PAMAM (≈200 nm) with MMP‐2 results in the formation of small‐sized particles (≈10 nm). After 4 h of MMP‐2 treatment, the small‐sized particles were further visually confirmed with transmission electron microscopy. Therefore, the enzyme‐sensitive strategy will open a new strategy in the further exploration of delivery systems for enhancing drug penetration and retention in the tumor site.

### 4.3 Light and redox‐responsive induced size shrinkage

**FIGURE 8:**
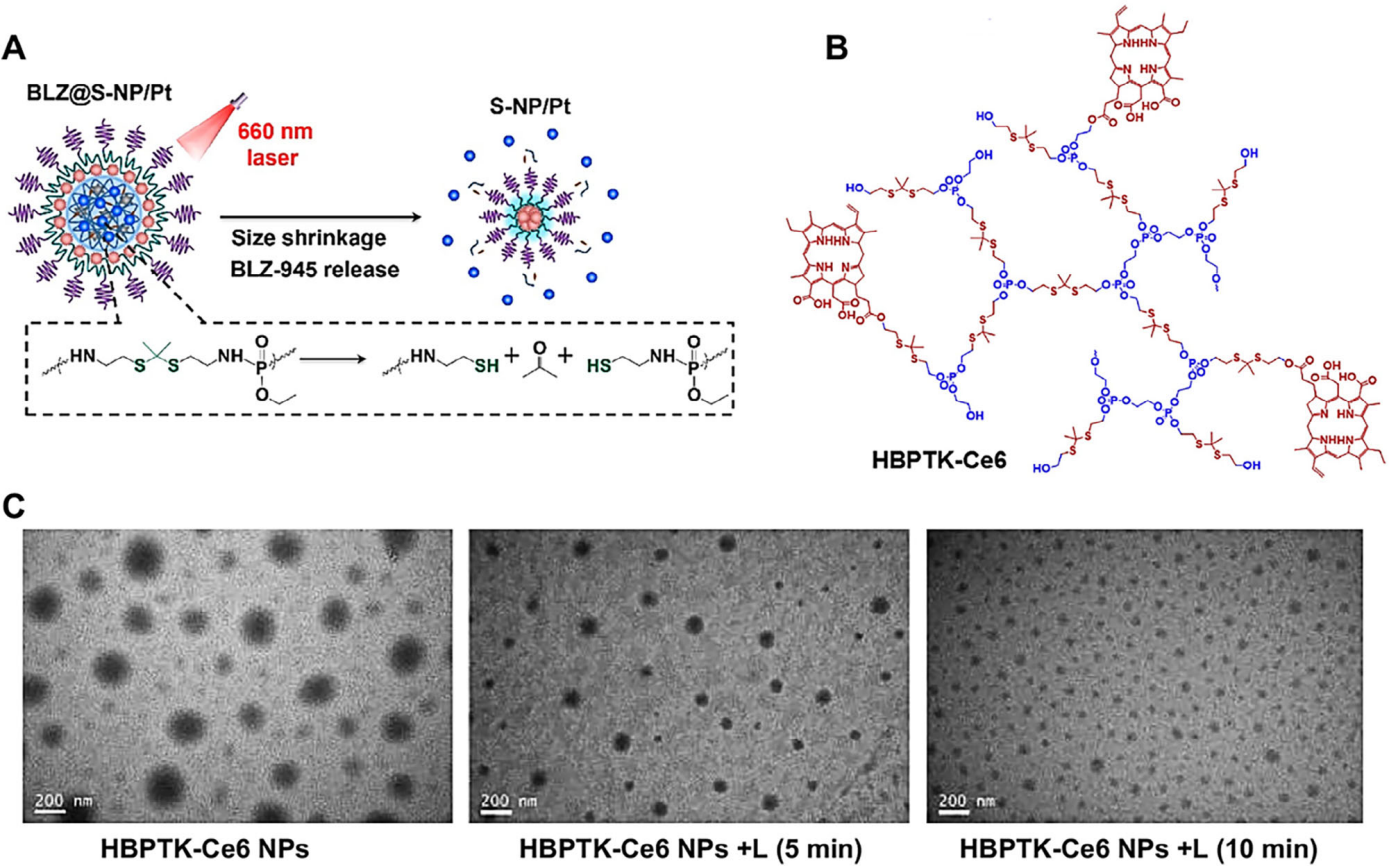
Light or redox‐triggered nanocarriers shrinkage strategies. (A) Schematic illustration of shrinkable nanoparticles BLZ@S‐NP/Pt for simultaneously targeting tumor cells and TAMs (tumor‐associated macrophages). Under 660 nm laser irradiation, BLZ@S‐NP/Pt could occur shrinkage and enhance penetration, simultaneously targeting “the soil and the seed” by differentially and precisely delivering BLZ‐945 and Pt(IV) prodrug to intended sites of M2‐TAM and tumor cell. Reproduced with permission.^[^ ^101^ ^]^ Copyright 2021, American Chemical Society. (B) The chemical structure of the light‐triggered ROS‐responsive hyperbranched polyphosphoester (HBPTK‐Ce6). (C) Representative TEM image of HBPTK‐Ce6 NPs upon 660 nm laser irradiation for different time. Reproduced with permission.^[^ ^102^ ^]^ Copyright 2019, Elsevier.

Since the GSH and ROS level is critical in tumor tissue and cytoplasm, it also can be employed in the design of size‐shrinkage nanocarriers.^[^ ^99^ ^]^ Additionally, exogenous stimuli, such as light, magnetic fields, and ultrasound, serve as potent tools in the design of size‐shrinkage nanocarriers.^[^ ^100^ ^]^ Wang and colleagues constructed a size‐shrinkable nanocarrier (BLZ@S‐NP/Pt, ≈70 nm) for the co‐delivery of anticancer drugs and immunotherapeutic agents, aiming to simultaneously target tumor cells and repolarize tumor‐associated macrophages.^[^ ^101^ ^]^ Upon light irradiation, the Ce6 (Chlorin e6) encapsulated within the particle core generated a significant amount of reactive oxygen species (ROS), which degraded the ROS‐responsive TK‐PPE core, triggering the size‐shrinkage of the particle and subsequent release of the immunotherapeutic agent BLZ945 for targeting the polarization of tumor‐associated macrophages (Figure 8A). The shrunken nanoparticles (≈30 nm) exhibit enhanced penetration capability and, further delivered the Pt drug deeper into the tumor tissue for effective tumor cell eradication, resulting in a pronounced antitumor effect. This photoactivated nanocarrier offers a viable approach to design co‐delivery systems, precisely delivering multiple therapeutic agents to their target cells and even to predetermined therapeutic sites. Yan et al.^[^ ^102^ ^]^ designed an ROS‐responsive nanoparticles based on amphiphilic hyperbranched polyphosphoester for drug delivery with light‐triggered size‐shrinkage propriety and enhanced tumor penetration (Figure 8B). Under light irradiation, the Ce6 within the nanoparticles generated a significant amount of ROS, which degraded the thioketal units in the nanoparticle core, leading to a size‐shrinkage process (Figure 8C). These size‐shrinkage nanoparticles could penetrate into tumor tissues more effectively, thereby enhancing therapeutic outcomes. Using IVIS (in vivo imaging system) imaging technology, the distribution and accumulation of nanoparticles in vivo were investigated, confirming excellent penetration ability of these shrunken nanoparticles.

## 5 DISINEGRATING STRATEGY

Nanocarrier‐mediated antitumor drug delivery, the therapeutic efficacy is often achieved through a series of in vivo transport processes, including prolonged blood circulation, tumor accumulation, penetration, cellular uptake, and rapid drug release.^[^ ^14^ ^]^ Among these, drug rapid release from nanocarrier therein stands as one of the pivotal factors for achieving optimal antitumor outcomes. Within the tumor microenvironment, nanocarriers can be designed to respond to intracellular stimuli, such as pH, enzymatic activity, redox conditions, hypoxia or extracellular light,^[^ ^103^, ^104^ ^]^ and radiotherapy, facilitating swift drug release.^[^ ^105^, ^106^, ^107^ ^]^ This ensures a sufficient drug concentration within a short timeframe to exert a therapeutic effect on the tumor, potentially overcoming multidrug resistance of tumor cells. Thus, the delivery of drugs to the tumor based on nanocarriers, followed by drug rapid release, is instrumental in improving the effective therapeutic potential of nanomedicines. By leveraging the several stimuli presenting in the tumor microenvironment, designing stimulus‐responsive disintegrative nanocarriers are designed to enhance drug release efficiency at the tumor site, amplifying the antitumor therapeutic effect.

### 5.1 Acidic pH‐induced disintegration

**FIGURE 9:**
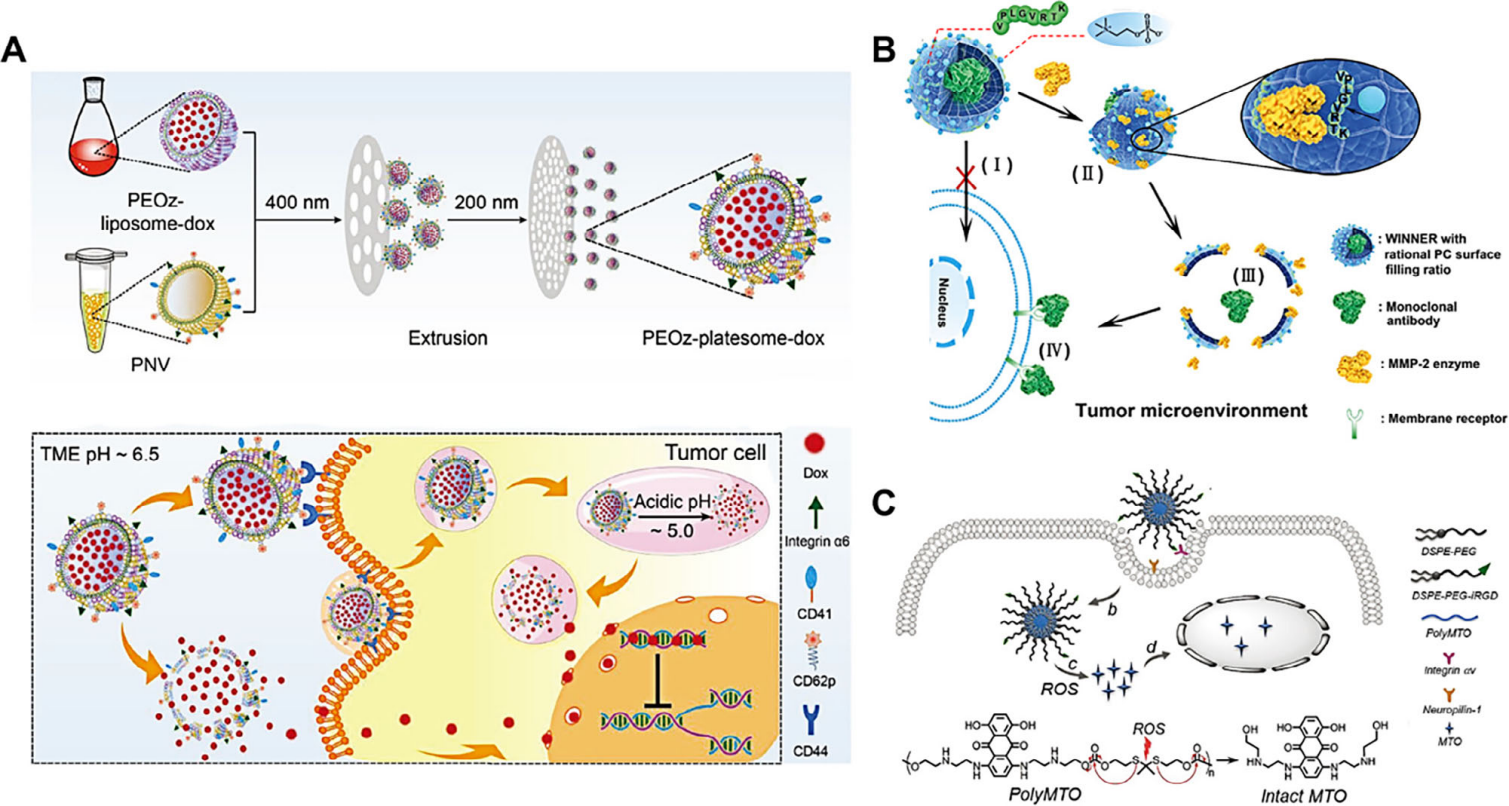
Nanocarriers disintegration strategies. (A) Schematic illustration of the preparation of PEOz‐platesome‐dox and its proposed antitumor mechanism. PEOz‐platesome‐dox was generated by coextrusion of PEOz‐liposome‐dox and platelet membrane nanovesicles (PNVs). After intravenous injection, PEOz‐platesome‐dox is expected to target the tumor through molecular interactions between platelet membrane and tumor cell substituents. Then, the incorporation of pH‐sensitive lipids into the platesome would allow its cargo (dox) to be rapidly released at the tumor site. Reproduced with permission.^[^ ^109^ ^]^ Copyright 2019, John Wiley and Sons. (B) Schematic design of WINNER with rational PC surface filling ratio for extracellular delivery of mAb for tumor suppression. WINNER has a functional core–shell structure: PC and enzyme‐responsive peptide engineered shell covers the inner protein drugs. The PC functional shell protects the inner protein drugs from cellular uptake. Subsequently, the extracellularly functional protein drugs are released from WINNER extracellularly upon the abundant MMP‐2 enzyme. Reproduced with permission.^[^ ^110^ ^]^ Copyright 2019, John Wiley and Sons. (C) Schematic illustration of the polyMTO‐based NP platform for targeted and deeply penetrating cancer therapy. Reproduced with permission.^[^ ^116^ ^]^ Copyright 2017, John Wiley and Sons.

Various pH‐sensitive materials have been employed to design efficient acidic‐responsive nanocarriers for enhanced therapeutic effect. Commonly, these acid‐responsive nanocarriers incorporate abundant pH‐responsive moieties. In the tumor environment, these pH‐sensitive moieties of nanocarriers undergo either scission of the linkage or protonation,^[^ ^108^ ^]^ resulting in altering the hydrophobic‐hydrophilic balance and inducing destabilization of the nanostructures, which eventually release the loaded active agents and exert antitumor therapeutic efficacy. Nie et al. introduced a novel biomimetic platesome nanocarrier system^[^ ^109^ ^]^ that was constructed by merging platelet membranes with functionalized synthetic liposomes (PEOz‐liposome‐dox), which exhibited enhanced tumor affinity and selectively release cargo in response to the acidic microenvironment of lysosomal compartments (Figure 9A). At pH 7.4, the PEOz‐platesome‐dox nanocarrier released approximately 58% of its loaded DOX content over 10 h. This release rate increased to 86% at pH 6.5 and reached nearly 100% within 4 h at pH 5.0. In contrast, the non‐pH‐responsive nanocarrier, PEG‐platesome‐dox, showed a maximum release of 62% over 10 h across all pH levels tested. Notably, the size of PEOz‐platesome‐dox expanded significantly after 1 h at acidic pH (5.0 or 6.5), but reduced in size after several hours, suggesting an acid‐triggered disassembly of the platesome structure. The engineered biomimetic platesomes offer a promising approach for targeted drug delivery to tumors. Their pH‐responsive design ensures the controlled release of drugs at tumor sites, enhancing therapeutic outcomes. The combination of pH‐triggered drug release and platelet membrane‐based camouflage contributes to the enhanced therapeutic efficacy of the platesome nanocarrier.

### 5.2 Enzyme‐induced disintegration

Leveraging the overexpressed enzymes in tumor tissues, enzyme‐responsive nanocarriers can be designed based on a series of enzyme‐responsive peptide sequences. This strategy enhances the delivery efficiency and therapeutic efficacy of anticancer drugs. Kang et al. designed a matrix metalloproteinase‐2 (MMP2)‐responsive proteinaceous drug delivery system that enables precise extracellular drug release (Figure 9B).^[^ ^110^ ^]^ By harnessing the high affinity of zwitterionic phosphatidylcholine (PC) and MMP2‐responsive peptides for co‐loading protein drugs, they developed the enzyme‐responsive nanocarrier, WINNER. The surface density of PC on WINNER, ranging from 50.5% to 58.3%, effectively reduced the interaction between the nanoparticles and the tumor cell membrane, thereby minimizing nanoparticle uptake by tumor cells. Furthermore, the overexpressed MMP2 in tumor tissues degraded the responsive peptides in the nanoparticles, triggering their disintegration in the extracellular environment and subsequent release of the encapsulated protein drugs. This study presents a versatile approach to design precise extracellular delivery platforms, unlocking the therapeutic potential of extracellularly targeted agents.

### 5.3 Redox‐triggered disintegration

As moderate levels of ROS play beneficial roles in cellular functions,^[^ ^111^ ^]^ excessive ROS can induce cellular damage.^[^ ^112^ ^]^ To counteract this oxidative stress, tumor cells upregulate the production of antioxidants, such as glutathione (GSH).^[^ ^113^ ^]^ Designing stimulus‐responsive nanocarriers tailored to the unique tumor microenvironment holds great promise, potentially enhancing therapeutic efficacy while minimizing adverse effects.^[^ ^114^, ^115^ ^]^

Xu and his colleagues designed an ROS‐responsive polyprodrug nanoparticles to avoid drug leakage during preparation, storage and blood circulation, meanwhile this nanoparticle could target tumor tissue and acquired better penetration.^[^ ^116^ ^]^ Such ROS‐responsive polyprodrug could self‐assemble into stable nanoparticles, providing high drug loading, active targeting tumor and enhanced penetration with surface‐encoded internalizing RGD (iRGD) and prolong blood circulation with outer PEG shell (Figure 9C). After internalization, the polyprodrug NPs could disintegrate with a chain‐breakage patterned triggered by intracellular high ROS level and release mitoxantrone (MTO) in the meanwhile, leading to significant inhibition of tumor cell growth. This targeted ROS‐responsive polyprodrug nanocarrier provided a new strategy to design stimulus‐triggered disintegrative nanocarrier for improving optimal properties of NP platform.

### 5.4 Other disintegration strategy

In addition to the abovementioned disintegration strategies, there are several other novel stimuli‐triggering approaches to make nanocarriers disintegrated in tumor regions. For example, Zhao et al. designed a novel hypoxia‐responsive human serum albumin (HSA)‐based nanosystem, denoted as HCHOA.^[^ ^117^ ^]^ This nanosystem was constructed through cross‐linking the hypoxia‐sensitive azobenzene group between photosensitizer chlorin e6 (Ce6)‐conjugated HSA (HC) and oxaliplatin prodrug‐conjugated HSA (HO), remaining stable under normal oxygen conditions with a size of 100–150 nm. In the hypoxic tumor microenvironment, the HCHOA nanosystem underwent rapid dissociation into ultrasmall HC and HO therapeutic nanoparticles (less than 10 nm), enhancing intratumoral penetration. Following dissociation, the quenched fluorescence of Ce6 in the HC nanoparticles could be recovered for bioimaging, and the production of singlet oxygen was increased due to enhanced photoactivity. This innovative strategy enabled combined photodynamic therapy and chemotherapy, demonstrating superior antitumor efficacy in vivo. Moreover, Chen et al. developed a nanovesicles containing gold nanoparticles tethered with irradiation labile linoleic acid hydroperoxide (LAHP) molecules and oxidation‐responsive poly(propylene sulfide)‐poly(ethylene glycol) (PPS‐PEG) polymers, which loaded with DOX in the vesicle's inner core, obtained Au‐LAHP‐vDOX.^[^ ^118^ ^]^ Upon irradiation, hydroxyl radicals (•OH) formed from LAHP trigger internal oxidation of PPS, transitioning it from hydrophobic to hydrophilic. This process leads to vesicle degradation and a burst release of drug cargo, resulting in synchronous chemoradiation. The approach demonstrated impressive anticancer efficacy both in vitro and in a subcutaneous mouse tumor model with a single injection and irradiation session.

## 6 SHAPE CHANGING STRATEGY

The engineering of nanocarriers with tumor‐specific morphological alterations, including fibrotic transformation, particle swelling, and phase transitions, can amplify tumor penetration, extend retention, evade immune clearance, and bolster tumor cell internalization, thereby elevating therapeutic efficacy. Specific morphologies of nanocarriers, such as fibrous structures,^[^ ^119^, ^120^ ^]^ facilitate enhanced penetration through the interstitial spaces of tumors owing to their elongated configurations, thereby achieving a more profound drug distribution. Post‐morphological transformation, these nanocarriers may exhibit prolonged retention within the tumor tissue, ensuring sustained drug release.^[^ ^121^ ^]^ Moreover, certain morphological transitions can augment the interaction between nanocarriers and cellular membranes, enhancing cellular uptake. Consequently, the strategic design of nanocarriers undergoing morphological changes at tumor sites presents a novel paradigm in cancer therapy, thus optimizing drug delivery efficacy, enhancing therapeutic outcomes, and minimizing adverse effects.

### 6.1 Shape remodel strategy

**FIGURE 10:**
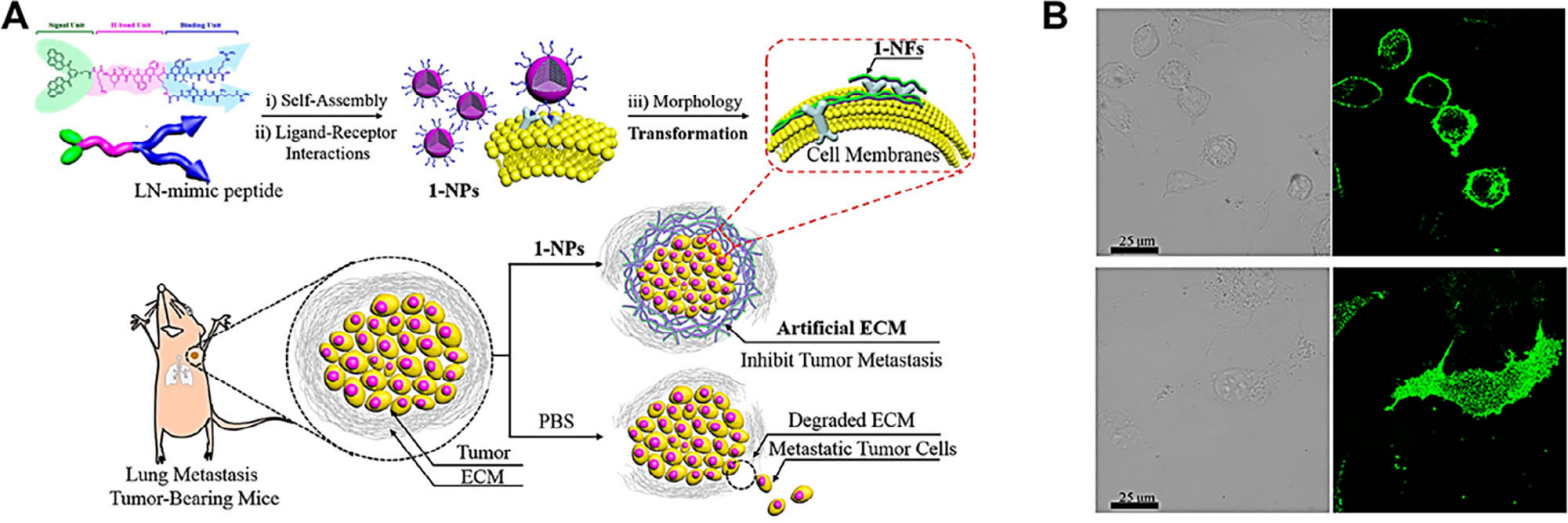
Nanocarriers fibrotic transformation strategy. (A) Schematic illustration of the Y‐type structure of peptide and the biomimic construction of AECM based on transformable 1‐NPs for high‐efficient inhibition of tumor invasion and metastasis. (B) CLSM images of MDA‐MB‐231 cells incubated with 1‐NPs (30 µm, up) and 2‐NPs (30 µm, down) for 2 h, respectively. Reproduced with permission.^[^ ^125^ ^]^ Copyright 2017, American Chemical Society.

Enhancing retention through the strategic design of nanomedicines represents a viable therapeutic strategy. Among various morphologies influencing nanomedicine retention, nanofibers stand out due to their exceptional retention capabilities.^[^ ^122^ ^]^ Nonetheless, their linear configuration poses challenges during circulation and distribution, as these nanomedicines are prone to sequestration in organs with intricate microvasculature, including the liver and lungs. A potential solution entails utilizing nanomedicines with an initial spherical morphology for optimal circulation and biodistribution, followed by a triggered morphological transition to fibrous structures to augment retention within the tumor milieu. Wang et al. proposed an “in vivo self‐assembly” strategy for in situ construction of nanofibers in vivo.^[^ ^123^, ^124^ ^]^ They developed a series of nanocarriers that could transform into nanofibers at tumor sites, which were based on the KLVFF β‐sheet peptide domain found in β‐amyloid (Aβ) peptide. For example, an artificial extracellular matrix (AECM) within tumor sites was constructed by leveraging a transformable Laminin (LN)‐mimetic peptide 1,^[^ ^125^ ^]^ denoted as BP‐KLVFFK‐GGDGR‐YIGSR, for the purpose of the inhibition tumor invasion and metastasis (Figure 10A). This mimetic peptide encompassed a bis‐pyrene (BP) unit, the KLVFF peptide motif, and a Y‐type RGD‐YIGSR peptide sequence. In aqueous solutions, this peptide self‐assembled into stable nanoparticles, termed 1‐NPs. Upon systemic administration, these nanoparticles targeted tumor sites where the peptide sequences on their surface interacted with receptors on tumor cells, subsequently undergoing a transformation into nanofibers (1‐NFs). CLSM observation shown that 1‐NPs treated MDA‐MB‐231 cells showed green fluorescence on cell surfaces, while 2‐NPs treated MDA‐MB‐231 cells showed strong green fluorescence intracellularly. These results implied that 1‐NPs could achieve in situ transformation from 1‐NPs to 1‐NFs and the inhibit the internalization of 1‐NPs by tumor cells, while 2‐NPs were internalized into cells, which was usually observed for other nanoparticles (Figure 10B). These nanofibers further intertwined to establish the AECM, effectively impeding tumor cell invasion and metastasis. Notably, 1‐NPs demonstrated potent inhibitory effects on the migration and invasion of highly metastatic cancer cells, specifically reducing the migratory and invasive capabilities of MDA‐MB‐231 breast cancer cells to 21.4% and 8.6%, respectively, and those of B16‐F10 melanoma cells to 58.5% and 59.5%, respectively. In vivo studies further revealed that the AECM remained stable at the tumor site for over 72 h, exhibiting inhibition rates of 82.3% and 50.0% for metastatic breast cancer and melanoma, respectively.

Lam et al. engineered a sophisticated supramolecular peptide with the sequence BP‐FFVLK‐YCDGFYACYMDV, termed TPM1.^[^ ^126^ ^]^ This peptide was composed of a bis‐pyrene moiety with aggregation‐induced emission properties, a reverse sequence polypeptide FFVLK derived from the β‐sheet‐forming peptide domain of β‐amyloid (Aβ) peptide, and a disulfide cyclic peptide YCDGFYACYMDV with specificity for human epidermal growth factor receptor‐2 (HER2) binding. In aqueous solutions, this supramolecular peptide self‐assembled into spherical peptide nanoparticles, where the BP and FFVLK domains constituted the hydrophobic core, and the YCDGFYACYMDV peptide formed a negatively charged hydrophilic corona. Upon systemic administration, these peptide nanoparticles preferentially accumulate in HER2 overexpressed (HER2+) tumors. Subsequent interaction of the peptide on the nanoparticle surface with the HER2 receptor on tumor cells could trigger an in situ transformation of the nanoparticles into a fibrillar structural network, inhibiting HER2 receptor dimerization and suppressing downstream cellular signaling as well as the expression of genes associated with proliferation and survival in the tumor cell nucleus. These deformable nanocarriers, which undergo in situ morphological transformation to form nanofibers at tumor sites, offer a novel strategy for optimizing the therapeutic efficacy of nanomedicine in cancer treatment.

Compared with the above extracellular shape remodeling strategies, intracellular shape remodeling strategies aimed to controllably manipulate to construct complex intracellular superstructures that with diverse topologies and biological functions.^[^ ^127^, ^128^ ^]^ Li et al. described a method involving intracellular enzyme‐catalyzed polymerization for the efficient synthesis of polypeptides and the in situ construction of topology‐controlled nanostructures.^[^ ^127^ ^]^ The authors demonstrated that the phase behavior and topological structure of polypeptides are determined by monomeric peptide sequences. They further investigated the relationship between polymerization dynamics and the temperature‐dependent topological transition in biological conditions. Linearly grown elastin‐like polypeptides were shown to be biocompatible, forming nanoparticles with significant molecular accumulation and retention effects, while 3D gel‐like structures with thermo‐induced multi‐directional traction interfered with cellular fates. These findings open avenues for the development of new nanomaterials in living subjects for biomedical applications.

### 6.2 Nanocarriers swelling strategy

**FIGURE 11:**
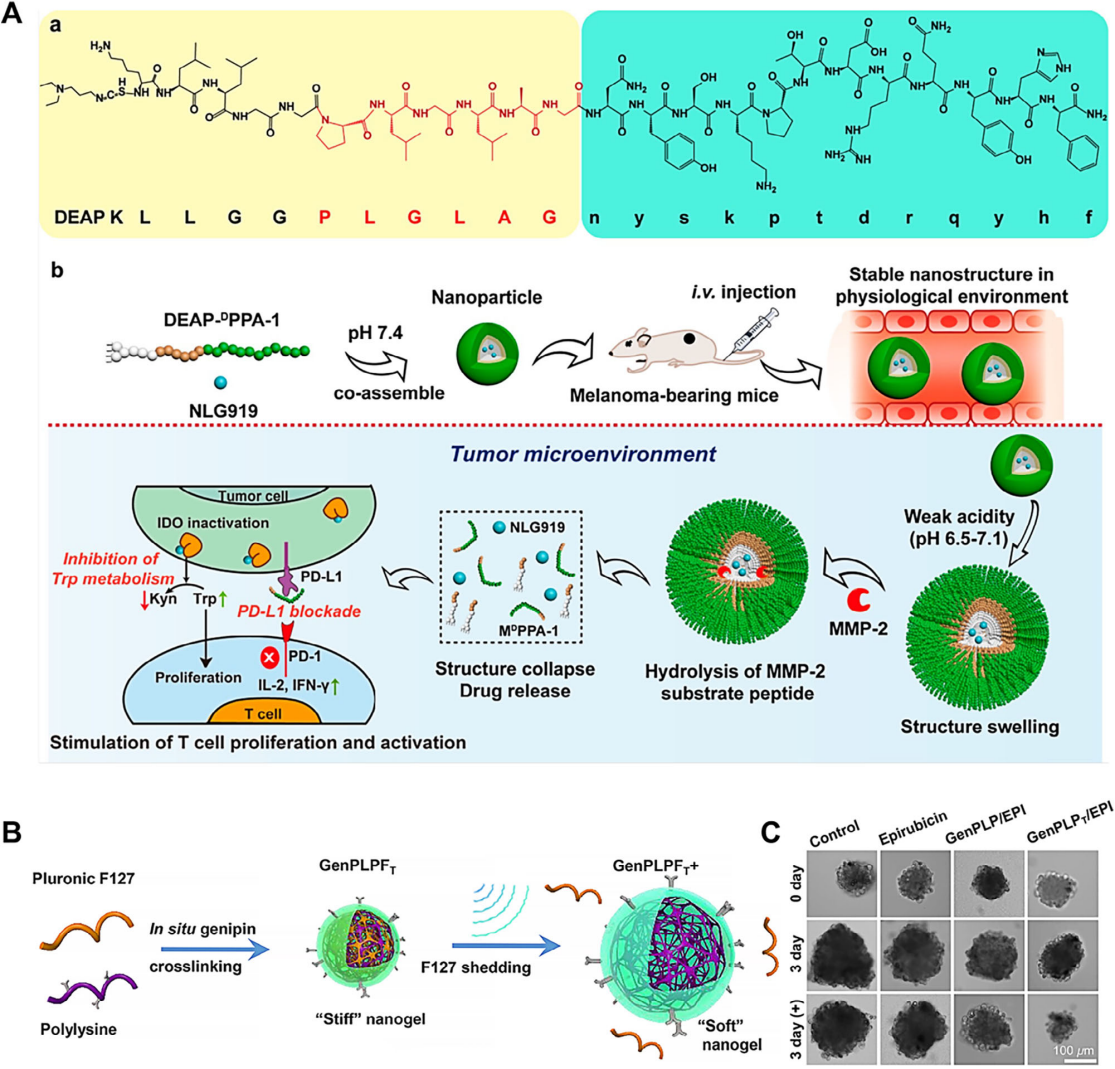
Nanocarriers swelling strategies. (A) The proposed antitumor mechanism of the NLG919@DEAP‐^D^PPA‐1 nanoparticle. DEAP‐^D^PPA‐1 and NLG919 co‐assemble into a nanoparticle and the hydrophobic core of the nanoparticle swells in response to tumor acidity, allowing MMP‐2 to access and hydrolyze its substrate peptide and leading to the complete dissociation of the nanostructure. Reproduced with permission.^[^ ^131^ ^]^ Copyright 2018, American Chemical Society. (B) Schematic illustration of stiffness‐tunable GenPLPFT/EPI for deep tumor delivery under external sonication, GenPLPFT could switch into softer and larger nanogel by pulse‐triggered pluronic F127 shedding. (C) Inhibition of cell sphere growth after treated with various epirubicin formulations. Reproduced with permission.^[^ ^132^ ^]^ Copyright 2022, American Chemical Society.

Stimulus‐responsive swell nanocarriers represent a subclass of morphologically transformable nanoparticles, facilitating rapid drug release at tumor sites.^[^ ^129^ ^]^ Upon specific stimuli, the polymer chains within these nanocarriers may undergo structural alterations or hydration, leading to nanoparticle expansion. For instance, pH‐sensitive nanocarriers might swell in acidic environments due to electrostatic repulsion between their polymer chains.^[^ ^130^ ^]^ As these nanocarriers swell, their loosened structure promotes enhanced drug release. Cheng and colleagues engineered a therapeutic peptide‐assembled nanoparticle capable of sequential dual‐stimuli responsiveness within the tumor extracellular matrix.^[^ ^131^ ^]^ This nanoparticle was tailored for tumor‐targeted delivery and on‐demand release of a short D‐type antagonist peptide 1 (^D^PPA‐1) 1 antagonist of programmed cell death ligand 1 (PD‐L1) and the indoleamine‐2,3‐dioxygenase (IDO) inhibitor, NLG919 (Figure 11A). The DEAP‐^D^PPA‐1 peptide was utilized to encapsulate NLG919 and self‐assembled into nanoparticles at pH 7.4. Upon systemic administration, these nanoparticles swelled in the mildly acidic environment of the tumor site. Subsequently, under the influence of matrix metalloproteinase 2 (MMP‐2), ^D^PPA‐1 was released to blocking the PD‐1/PD‐L1 checkpoint. Then, the nanoparticles continued to expand, disintegrate and release NLG919 for inhibiting the IDO enzyme, thereby stimulating T‐cell proliferation and activation.

Du and colleagues constructed an ultrasound‐responsive peptide nanogel, termed GenPLPF,^[^ ^132^ ^]^ which allowed for the modulation of gel particle stiffness and size, thereby enhancing tumor penetration and facilitating rapid drug release (Figure 11B). This stable nanogel particle was obtained by co‐assembling polylysine and Pluronic F127, and crosslinked by genipin, a natural cross‐linker derived from gardenia fruit, to form stable nanogel structure. Further, the nanogels were loaded with ICAM‐1 antibodies and chemotherapeutic agents, obtained the drug‐loaded nanoparticle GenPLPF_T_/EPI. Under external ultrasonic stimulation, Pluronic F127 dissociated from the nanogel leaded to an increasing cavitation volume of nanoparticle. This process allowed for much more enzyme and solvent infiltration into the nanogel, causing the GenPLPF to swell from 329 to 516 nm. In the meanwhile, its Young's modulus experienced a significant reduction from 336.78 to 3.93 kPa, amplifying the deformability of nanogels and decreasing its stability, thus promoting rapid drug release. The in vitro growth of 3D cell model (≈3.00 × 10^5^ µm3) treated with various epirubicin formulations with or without sonication also revealed the GenPLPF_T_/EPI exhibited the best tumor inhibition effects with sonication (Figure 11C). Additionally, the ultrasound application can reduce the interstitial pressure within tumor tissues, to promote blood circulation and enhance tumor penetration, resulting in acquired better antitumor efficacy. This strategy provided a new way to design deformable nanocarriers for enhanced tumor penetration and treatment.

### 6.3 Phase transition/separation strategy

Phase‐transition/separation nanocarriers can be designed to respond to either endogenous or exogenous stimuli, such as pH, temperature, enzymes, light, and magnetic fields.^[^ ^133^ ^]^ Upon encountering these stimuli, the physical or chemical properties of the nanocarriers undergo alterations or different components within the nanocarriers may undergo phase separation, leading to alterations in their morphology and functionality. Typically, these nanocarriers are composed of polymers or lipids that possess specific responsiveness. Under certain conditions, these materials can transition from one physical state (e.g., solid or gel‐like) to another (e.g., liquid), facilitating controlled drug release. For example, Zhang et al. functionalized single‐walled carbon nanotubes (SCNTs) and multi‐walled carbon nanotubes (MCNTs) with temperature‐sensitive peptide lipids (PL) and sucrose laurate (SL),^[^ ^134^ ^]^ resulting in bifunctional delivery systems with exceptional thermosensitive photothermal properties, termed SCNT‐PS and CNTs‐PS, respectively. CNTs‐PS could adsorb siRNA therapeutics through electrostatic interactions. Upon light exposure, the CNTs generated heat to induce a phase transition in the lipids on the CNTs surface, leading to their detachment and triggering the rapid release of anti‐survivin siRNA. This approach synergistically was combined with photodynamic therapy and gene therapy, achieving promising antitumor efficacy.

## 7 OTHER DEFORMABLE NANOCARRIERS

Beyond the aforementioned morphological transformations of nanocarriers, other deformable processes of functional nanocarriers also can undergo in the tumor microenvironment, including twisting, rotation, or pore formation.^[^ ^135^ ^]^ These modifications enable nanocarriers to better adapt to the intricate conditions of the tumor site, enhancing the delivery efficiency of anti‐tumor drugs. For example, Zhu et al. designed a magnetic‐driven deformable amoeba‐like nanorobot based on a flowable polyphosphoester core.^[^ ^136^ ^]^ Under the guidance of a magnetic field, the nanoparticles undergo deformation and twisting, enabling them to overcome multiple physiological barriers in vivo drug delivery, thereby enhancing drug delivery efficiency and therapeutic efficacy.

## 8 CONCLUSION AND OUTLOOK

In recent decades, the field of nanocarriers has seen remarkable advancements, especially in drug delivery and biomedical applications. However, challenges persist, including optimizing drug loading, ensuring precise drug targeting, reducing off‐target biodistribution, and addressing biocompatibility issues. To overcome these obstacles, recent research has focused on the design of deformable nanocarriers. These nanocarriers are crafted to undergo structural and functional changes in response to specific stimuli within the tumor microenvironment, enhancing drug delivery efficiency and therapeutic results. The distinctive characteristics of deformable nanocarriers represent a paradigm shift in drug delivery strategies. Their ability to provide precise and efficient drug delivery solutions holds immense potential for elevating therapeutic outcomes while concurrently mitigating undesirable side effects, including amplified bioavailability, precision in targeted delivery, fine‐tuned drug release dynamics, and adaptability to biological hurdles. For example, nanocarriers that adapt to tumor microenvironmental factors, such as pH, temperature, or enzymes—using approaches like cross‐linked aggregation, size shrinkage, particle disintegration, morphological transformation, and other deformation techniques—have shown promise in both preclinical and clinical stages.

Deformable nanocarriers, distinguished by their adaptability to physiological conditions, exhibit significant potential for clinical translation in drug delivery. These nanocarriers, encompassing liposomes and polymeric nanoparticles, dynamically alter their shape in response to environmental cues, facilitating precise drug delivery to target tissues, particularly in tumors. Their responsiveness to factors like pH or temperature enables controlled drug release. Deformable nanocarriers surmount biological barriers, enhancing drug penetration and mitigating off‐target effects. Ongoing research emphasizes optimizing design for clinical applications, supported by preclinical studies showcasing their effectiveness in delivering diverse therapeutic agents. Despite challenges, the promising preclinical data indicates a transformative role for deformable nanocarriers in personalized and targeted medicine.

Though great progress has been made in deformable nanocarriers, there are still many challenges that hider the clinical translation of these nanocarriers, including encompass ensuring biocompatibility, achieving precise delivery while minimizing off‐target effects, navigating biological barriers like the blood‐brain barrier, maintaining stability under physiological conditions, and addressing potential long‐term toxicity issues. Furthermore, challenges related to scalable production and regulatory constraints can hinder their clinical progression. In this regard, researchers are directing their efforts towards the design of biodegradable nanocarriers, enhancing targeting ligands, adopting multifunctional designs to traverse biological barriers, undertaking thorough pre‐clinical toxicity assessments, and fostering close collaborations with regulatory bodies to expedite the approval pathway. Looking forward, the convergence of nanotechnology, biology, and medicine will likely steer nanocarrier development towards more personalized and sophisticated designs that cater to complex biomedical needs. Simultaneously, we can expect a surge in nanocarrier innovations that advance to clinical trials and, eventually, commercial availability.

## CONFLICT OF INTEREST STAEMENT

The authors declare no conflicts of interest.
